# Semiautonomous Robotic Manipulator for Minimally Invasive Aortic Valve Replacement

**DOI:** 10.1109/TRO.2023.3315966

**Published:** 2023-10-09

**Authors:** Izadyar Tamadon, S. M. Hadi Sadati, Virginia Mamone, Vincenzo Ferrari, Christos Bergeles, Arianna Menciassi

**Affiliations:** Faculty of Engineering Technology, University of Twente, 7522 NB Enschede, The Netherlands, and also with the BioRobotics Institute, Scuola Superiore Sant’Anna, 56025 Pontedera, Italy; Robotics and Vision Department in Medicine Lab, School of Biomedical Engineering & Imaging Sciences, King’s College London, SE17EU London, U.K.; Department of Computer Science and the EndoCAS Center for Computer-Assisted Surgery, University of Pisa, 56124 Pisa, Italy; Department of Computer Science and the EndoCAS Center for Computer-Assisted Surgery, University of Pisa, 56124 Pisa, Italy; Robotics and Vision Department in Medicine Lab, School of Biomedical Engineering & Imaging Sciences, King’s College London, SE17EU London, U.K.; BioRobotics Institute, Scuola Superiore Sant’Anna, 56025 Pontedera, Italy

**Keywords:** Aortic valve surgery, minimally invasive surgery (MIS), robotic surgical endoscopy, surgical manipulator control, surgical navigation

## Abstract

Aortic valve surgery is the preferred procedure for replacing a damaged valve with an artificial one. The ValveTech robotic platform comprises a flexible articulated manipulator and surgical interface supporting the effective delivery of an artificial valve by teleoperation and endoscopic vision. This article presents our recent work on force-perceptive, safe, semiautonomous navigation of the ValveTech platform prior to valve implantation. First, we present a force observer that transfers forces from the manipulator body and tip to a haptic interface. Second, we demonstrate how hybrid forward/inverse mechanics, together with endoscopic visual servoing, lead to autonomous valve positioning. Benchtop experiments and an artificial phantom quantify the performance of the developed robot controller and navigator. Valves can be autonomously delivered with a 2.0±0.5 mm position error and a minimal misalignment of 3.4±0.9°. The hybrid force/shape observer (FSO) algorithm was able to predict distributed external forces on the articulated manipulator body with an average error of 0.09 N. FSO can also estimate loads on the tip with an average accuracy of 3.3%. The presented system can lead to better patient care, delivery outcome, and surgeon comfort during aortic valve surgery, without requiring sensorization of the robot tip, and therefore obviating miniaturization constraints.

## I Introduction

Aortic valve disease is the most common form of heart disease affecting elderly patients. In severe cases, a surgical procedure may be needed to replace the damaged valve due to calcification with an artificial one [1]. Cardiac surgeons evaluate the individual’s health condition and risk factors to determine the best replacement surgery [2]. The surgical procedure can be performed in several ways but can be grouped as follows.

1) Surgical aortic valve replacement (SAVR): This is the most accurate procedure, with wide access to the heart and fixing an artificial valve with sutures. However, it requires wide exposure in the sternum, which might not be suitable for elderly or high-risk patients [3], [4].
2) Minimally invasive aortic valve replacement (MIAVR): It is performed with partial direct exposure (30–40 mm) to allow removing the calcific valve and accurate positioning of the prosthesis through elongated and rigid tools [5]. This procedure can benefit low-to-moderate risk patients, although a number of complications are directly related to the rigidity of delivery tools, vision restrictions, and limited sensing [6].
3) Transcatheter aortic valve replacement (TAVR): It is a procedure to deliver an aortic valve through a safe incision, such as the transfemoral access [7]. This replacement surgery is mainly performed in high-risk patients who might not tolerate SAVR. Paravalvular leakage and the need for a cardiac pacemaker are common issues in TAVR because of difficulties in achieving accurate valve positioning, orientation, and uniformity. Therefore, a controllable and flexible robotic system that minimizes thoracic trauma and accurately releases the prosthesis can significantly improve surgical outcomes.

Research in robotic valve replacement started with the use of the Da Vinci surgical system in 2005 [8] and continued in 2010 with the complete excision of diseased native aortic valve leaflets and the accurate placement of a Sorin Perceval artificial valve [9]. Although robotic cardiac surgery has demonstrated several benefits, such as smaller incisions, motion scaling, and enhanced visualization [10], [11], [12], [13], the introduction of the Da Vinci robot into cardiac surgery has been slow, mainly because its grasping solutions are not designed for holding and manipulating the artificial valve [14], [15]. To overcome the abovementioned limitations, dedicated robotic systems were introduced for aortic heart valve surgery, albeit with rigid sheath and limited actuation [16].

An actuated delivery sheath was developed in [17] with 2 degrees of freedom (DoFs) bending and 1-DoF insertion. It demonstrated preliminary positive results for valve positioning in transapical aortic valve implantation. However, to accurately position the valve, flexible robotic solutions for MIAVR require extra DoFs, such as valve rotation and translation, to properly adjust the valve within the anatomy, which is basically a “hit or miss” task: a mismatch in position or orientation results in suboptimal early and long-term leakages [18].

In order to increase the accuracy, intraoperative magnetic resonance imaging (MRI) technologies [16], ultrasound tracking [17], and fluoroscopic imaging [19], [20] have been proposed to guide a valve delivery procedure with an external tracking equipment. Flexible robotic systems with integrated visualization that can close the loop in the system controller could increase the system precision, as well as presented in other application scenarios [21], [22], [23].

We studied MIAVR via a right anterior minithoracotomy as a reference treatment [18]. In this surgery, a 30–35 mm long incision is created in the chest as the first step. Then, the heart–lung machine is planted, the heart is stopped, and the calcified valve is removed. Among the commercially available sutureless heart valves, Sorin Perceval S is considered as an ideal prosthesis due to its large availability and promising long-term outcome [24], [25].

### A Related Works

We introduced the concept of a flexible articulated manipulator with endoscopic vision and flaps on the tip to perform a right minithoracotomy MIAVR via a transaortic approach [26]. In [27], the reported cable-driven articulated manipulator was manually actuated by a technique that minimized cable slackening. Also, the classic constant curvature (CC) modeling was implemented to assess the manipulator’s workspace in terms of repeatability and reversibility. In 2021, the ValveTech robotic platform was evolved in terms of visualization, which was obtained by three endoscopic cameras mounted around the robot’s distal end [28]. An inner mechanism (called the *introducer*) provided valve translation, axial rotation, and valve expansion following the surgeon’s commands. The manipulator was placed on a chest phantom, including a 3-D printed rib cage and silicone vessels, by a passive arm and controlled by open-loop controllers. The systems’ functionality in a simulated in vitro surgical scenario was positively validated by ten cardiac surgeons.

The system presented in [28] has unique features when compared to state-of-the-art robotic prototypes presented in [16] and [17]: stabilizing flaps to maintain the manipulator in position; two-stage valve expansion allowing small adjustments; and all the required 5-DoFs to position, orient, and release the valve (see Part I of the Supplementary Material). Our robotic approach is an attractive solution for MIAVR, being able to increase the quality of the intervention for surgeons and benefit patients with faster recovery. The robot may in particular benefit patients with complicated anatomies that would otherwise require an open-heart approach (SAVR) [29]. However, we found that through pure teleoperation, the surgeon was not able to safely approach the manipulator to the anatomical site with minimum contacts and was not able to accurately center the tip in a confined space, thus resulting in damage to the vessels and mismatches upon release.

### B Contributions

**Fig. 1:**
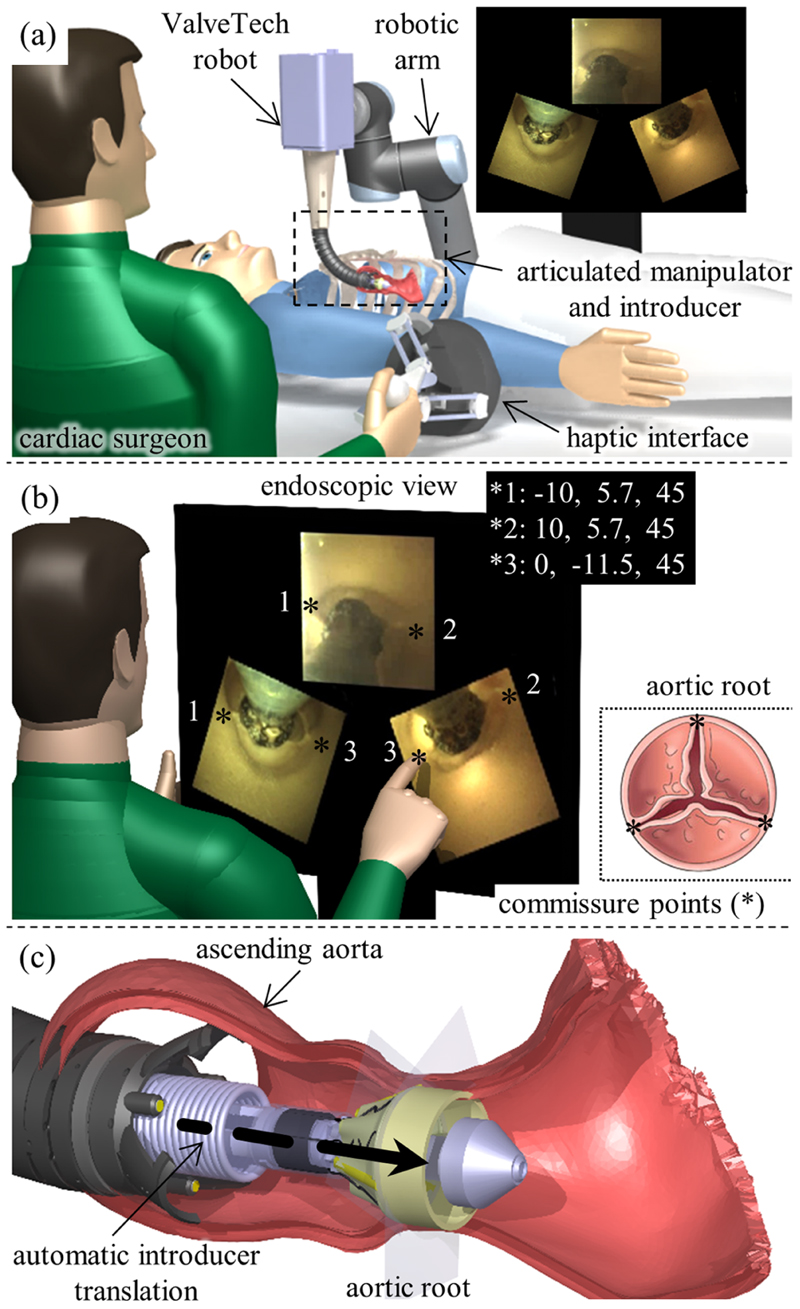
Schematic of MIAVR by the proposed robot. (a) Illustration of the procedure operated by a surgeon with the help of an endoscopic view and haptic interface to reach the intervention site (phase I). (b) Exploring and selecting the commissure points (i.e., the space between each anchored leaflet and the aortic wall) in the endoscopic views by the surgeon (phase II). (c) Automatic centering of the tip and introducer translation to the expansion site based on image data (phase III).

In this article, we present safe, semiautonomous navigation of the ValveTech robot prior to valve implantation. We describe a new strategy for initial planning, base positioning, and safe guiding of the cable-driven articulated manipulator through the body. In the first phase, the manipulator is haptically controlled to reach the ascending aorta by monitoring the forces on its tip for a full sensing of the procedure. This feature helps the surgeon safely guide the robot with minimum contact with the surrounding vessels or organs [see Fig. 1(a)]. Endoscopic views are utilized for efficient automatic control of the manipulator and introducer. In the second phase, the identical anatomical landmarks are selected by the surgeon in at least two views [see Fig. 1(b)]. Then, during the third phase, the manipulator is automatically centered in the aortic root, and the introducer automatically translates the valve to the expansion site for a correct delivery [see Fig. 1(c)]. By means of this new controller, the manipulator is efficiently positioned in the confined space, and delivery accuracy can be increased through accurate motion in a restricted workspace.

In more detail, this article contributes

1) an autonomous safe insertion planning;
2) a force observer that estimates the load on the robot tip;
3) forward and inverse mechanics (FM and IM) of the cable-driven manipulator based on hybrid force/position approaches;
4) an automatic navigation that uses anatomical coordinates and robot IM for closed-loop position control.

We demonstrated robot performances and valve positioning accuracy in a series of benchtop experiments, as well as in a simulated surgical scenario employing a realistic phantom.

The article is organized as follows. Section II describes the target surgical scenario and provides an overview of the whole system design, including the manipulator’s modeling/control and the surgical navigator. Section III details the experimental procedures (i.e., each individual unit separately and the full robotic system in its workspace). Moreover, the valve delivery procedure in the simulator and error measurement methods in comparison with the ideal position are presented. Section IV reports the experimental results, followed by their discussion in Section V. Finally, Section VI concludes this article. Appendix A includes the forward kinematics (FK) based on CC assumptions. The FM for shape observation and a generic hybrid shape control and force observer framework are discussed in Appendix B.

## II System Description

In this section, we initially present the intended operation that the ValveTech robot is planned to perform. The hardware of the system is briefly presented, whereas the software is presented in detail.

### A Description of the Surgical Operation

**Fig. 2:**
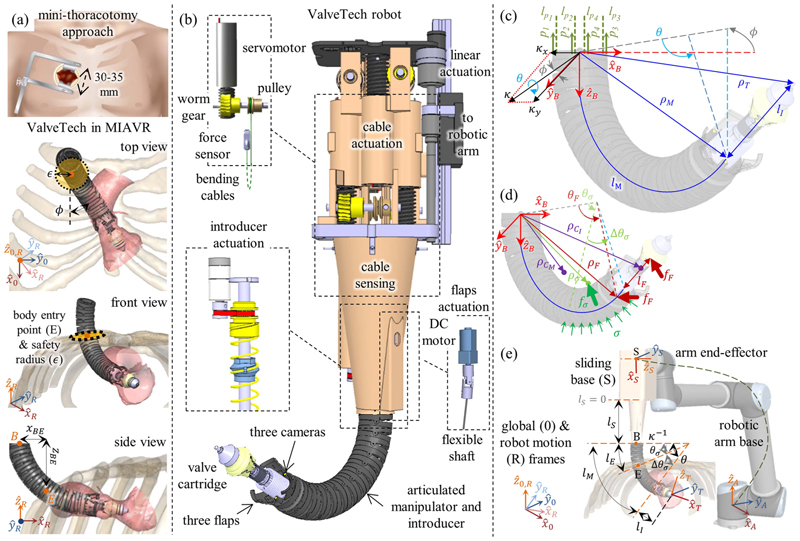
System overview. (a) Geometrical considerations for robotic minithoracotomy MIAVR. (b) Robot overview and its structures showing cable actuation and sensing principle, introducer, and flap actuation. (c) Manipulator in a bent configuration and its kinematic modeling principle. (d) System mechanics and external load modeling principle. (e) Robot motion planning to access the aortic valve.

The robot is intended to operate as sketched in Figs. 1 and 2(a). Coarse positioning and system suspension are achieved by a standard robotic arm, which maintains the cable-driven manipulator in proximity to the surgical incision. Then, the cable-driven manipulator is guided by the surgeon to be positioned 3–4 cm behind the valve delivery site based on the view provided by the endoscopic cameras. When the manual positioning is finalized, the anatomical landmarks consisting in the commissure points [see Fig. 1(b)] are identified, and the introducer is autonomously moved to the implantation site by the system controller. After that, to achieve perfect matching of the valve with the commissures, the surgeon adjusts the valve rotation along its main axis. Finally, valve expansion is commanded for full attachment to the annulus wall. During the procedure, valve rotation and expansion are manually actuated to maximize safety in the operation. The introducer can be retrieved from the expanded valve and the robot can be completely retracted, again under image guidance.

### B Robot Design

The complete system design has already been presented [27], [28]. Therefore, here it is only briefly summarized by highlighting improvements in the introducer actuation mechanism.

The ValveTech robot was designed in collaboration with cardiac surgeons, in accordance with clinical considerations, and in line with Perceval S valve characteristics. The manipulator’s length is 140 mm and can cover a workspace of 250 × 250 × 80 mm^3^. The manipulator’s internal diameter is based on crimped valve diameter (i.e., 21 mm), and the external diameter is based on the opening incision on the chest (i.e., 30–35 mm).

The ValveTech robot structure consists of the following four main sections:

1) flexible articulated manipulator;
2) cable actuation and sensing;
3) cameras and flaps;
4) valve introducer [see Fig. 2(b)].

#### 1) Flexible Articulated Manipulator

The manipulator was designed as a chain of 26 hollow links (*ϕ*_out_ = 28 mm, *ϕ*_in_ = 23 mm) connected with pin joints. The links have 5° cuts on their edges to achieve global 2-DoFs bending up to 120°.

#### 2) Cable Actuation and Sensing

The manipulator’s bending is obtained by actuating two pairs of cables. The bending cables were routed to have one end connected to a force sensor and the other end to an actuation motor [see Fig. 2(b)]. Each bending cable was actuated by a brushless dc servomotor (Faulhaber 2250BX4, Germany) through a 1:10 ratio worm gear and a pulley (*ϕ* = 13 mm). The other end was fixed to a force sensor (Futek, LSB200, USA) capable of measuring up to 44.5 N. The four actuation subsystems (each consisting of servomotor, worm gear, and pulley) were assembled to obtain a compact (10 × 10 × 15 cm^3^) actuation unit; the sensors were correspondingly integrated into a sensing unit located close to the manipulator’s proximal section. Additionally, between the connection of the actuation unit and the robotic arm, a linear rail and ball screw (Faulhaber BS22-1.5, Germany) actuator with an 80-mm stroke was positioned to provide linear insertion into the body at a speed of 1 mm/s.

#### 3) Cameras and Flaps

Cameras were embedded to help surgeons explore the surgical area with direct endoscopic vision (relevant in phase I) and as feedback to close the control strategy (phase II). Three chip-on-tip cameras were laterally embedded on the top link of the manipulator at 120° angular shifted positions, and their signal wires passed through the manipulator links. FISCam (FISBA, Switzerland) cameras were selected owing to their small diameter (1.95 mm), wide diagonal field of view, and visual depth of field of up to 5 cm. More specifically, the FISCam operates at 30 frames per second with a 400 × 400 pixel resolution (*C_H_* × *C_V_*) and a 60° horizontal and vertical field of view (FOV_*H*_ and FOV_*V*_).

The nominal diameter of the ascending aorta is usually less than 40 mm [30]. Therefore, three flaps were designed to stabilize the manipulator during introducer positioning by damping small delivery vibrations and to prevent imaging occlusion from potentially aortic wall collapsing [26].

#### 4) Introducer

**Fig. 3:**
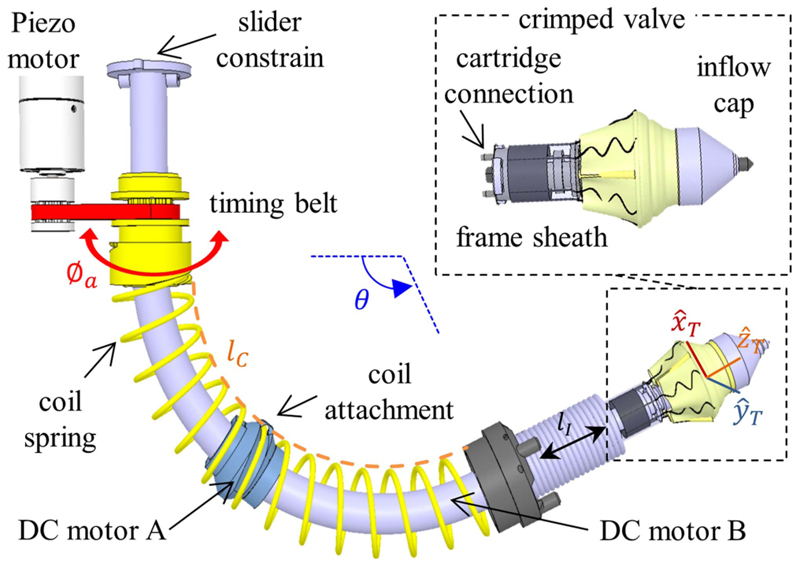
Introducer mechanism for converting the spring rotation to linear translation of the valve. This valve is crimped and held inside the manipulator by a custom cartridge, and it is charged in the robotic introducer before entering the body.

Our introducer’s design is unique as it provides translational motion without affecting the manipulator’s bending. The introducer, which is in the free lumen of the manipulator, includes a rotatable coil spring (*ϕ* = 21.6 mm, *ϕ*_coil_ = 1.6 mm), a coil attachment joint, and a slider constraint. A motor (PiezoMotor, LR17, Sweden) actuates a pair of pulleys and a timing belt to ultimately rotate the coil spring. This modification was devised to facilitate the camera wires sliding during manipulator bending. By previous spur gear actuation [28], a camera wire was blocked in its channel, leading to hamper the bending and data wire breaking. The introducer provides axial translational motion of up to 60 mm with a maximum linear speed of 0.5 mm/s. Two dc gear motors (MFA/COMO Drills, Series 951D, U.K.) for valve rotation around the main axis (A) and valve expansion (B) (see Fig. 3) have been considered in this unit. The introducer has a slider (60 mm in length) on the backside, and a cartridge connection on the distal tip. On the distal tip of the sliding chain, a stainless-steel tension spring (*ϕ* = 16.5 mm, *ϕ*_coil_ = 1.4 mm) is placed to satisfy the flexibility of the system and constrain the introducer to follow the proper orientation of the head plane (maximum ±3° error while the introducer is completely out). A rotational knob switch drives the dc gear motor A of the introducer, causing the cartridge to rotate clockwise or counterclockwise. A button on a custom breadboard runs motor B for the first expansion stage on the inflow cap and the second expansion stage on the frame sheath.

The cartridge can hold bigger Perceval valves if it is enlarged in a straightforward manner, but the introducer should go through a major redesign to be matched with other artificial valve types.

### C System Mechanics for Force/Position Control/Observation

The robot tip position is modeled based on a modified singularity-free CC model to accommodate the effects of external, body, tendon, and internal frictional forces based on the principle of virtual work (PVW) (see Section II-C1) [31]. The singularity-free representation of CC utilizes curvature components *κ*_*x*|*y*_ instead of the standard curvature and bending plane polar angle [32] to solve the singularity issues associated with the robot’s straight configuration.^1^ By combining the information about tendon length changes together with tension values, we set up a simple framework for hybrid tip force/position observation based on the system’s FK and hybrid force/position control based on the system’s inverse kinematics (IK) (see Section II-C4). During the intervention, only the manipulator bending and tip introducer translation motions are considered as the system’s DoFs for position tracking and force observation. The base sliding motion, which is utilized for automated safe motion planning during the robot insertion into the patient’s body, is discussed in Section II-D.

#### 1) Forward and Inverse CC Kinematics

In the CC assumptions, the curve can be described by three variables [*ϕ*, *κ*, *l_M_*], where *κ* is the curvature, *ϕ* is the bending plane polar angle, and *l_M_* is the backbone length (fixed in our case). Fig. 2(c) presents the robot diagram. A singularity-free representation of CC is possible based on the set [*κ_x_*, *κ_y_*, *l_M_*], where *κ*_*x*|*y*_ are the curvature components along the local frame *x*- and *y*-axis. Then, we have $\kappa =\sqrt{\kappa_{x}^{2}+\kappa_{y}^{2}}$ and *ϕ* = tan^−1^(*κ_x_*/*κ_y_*), which results in a deterministic solution (*ϕ* = 0) for the robot straight configuration based on an IK formulation. The introducer unit extends along the manipulator tip tangent and up to a given length of *l_I_* (introducer length).

The relations that constitute the CC-based FK map of the manipulator are presented in Appendix A. The system states are *q* = [*κ_x_*, *κ_y_*, *l_I_*], where *θ* = *κl_M_* and the manipulator length *l_M_* is constant. The tip positions of the manipulator *ρ_M_* and introducer *ρ_T_* are given as follows:

(1) $$\begin{array}{l} \rho_{M}=\frac{1}{\kappa }.\left[\begin{array}{c} \cos(\phi )(-\cos(\theta )+1)\\ \sin(\phi )(-\cos(\theta )+1)\\ \sin(\theta ) \end{array}\right],\\ \rho_{T}=\frac{1}{\kappa }.\left[\begin{array}{c} \left[\cos(\phi )\left(l_{I}\kappa \sin(\theta )-\cos(\theta )+1\right)\right]\\ \sin(\phi )\left(l_{I}\kappa \sin(\theta )-\cos(\theta )+1\right)\\ \left(l_{I}\kappa \cos(\theta )+\sin(\theta )\right) \end{array}\right] \end{array}$$

where *T*_*M*|*T*_ are the manipulator and introducer tip 4 × 4 homogeneous transformation matrices, as shown in Appendix A.

The subscript CC denotes an IK solution based on CC and ignores external loads. The IK of the robot can be found based on the tip position vector *ρ_T_* and by solving (2) for *θ*_CC_. Equation (2) is a nonlinear geometrical relation to point the robot tip toward the target tip position *ρ_T_* in the bending plane with *ϕ*_CC_ = tan^−1^(*ρ_T_y__* / *ρ_T_x__*)

(2) $$\left(\frac{\pi }{2}-\theta_{\text{CC}}\right)-\tan^{-1}\left(\frac{\rho_{T_{y}}-\frac{l_{M}\sin\left(\theta_{\text{CC}}\right)}{\theta_{CC}}}{\rho_{T_{x}}-\frac{l_{M}\left(1-\cos\left(\theta_{\text{CC}}\right)\right)}{\theta_{\text{CC}}}}\right)=0.$$

As a result, we have *κ*_CC_ = *θ*_CC_/*l_M_*, *κ*_CC_*x*__ = *κ*_CC_ sin(*ϕ*_CC_), and *κ*_CC_*y*__ = *κ*_CC_ cos(*ϕ*_CC_) as the system states resulting from CC IK. Then, *l*_*I*CC_ is the distance between the manipulator tip and the target, which is given as follows:

(3) $$l_{I\text{CC}}=\sqrt{{\left(\rho_{T_{y}}-\frac{\sin\left(\theta_{\text{CC}}\right)}{\kappa_{\text{CC}}}\right)}^{2}+{\left(\rho_{T_{x}}-\frac{1-\cos\left(\theta_{\text{CC}}\right)}{\kappa_{\text{CC}}}\right)}^{2}}.$$

The real system states [*κ_x_*, *κ_y_*] (or alternatively, [*θ*, *ϕ*]) slightly deviate from the above CC states due to the system body and external loads and will be derived based on the system mechanics (see Section II-C3).

The initial lengths for the four tendons *l_P_i__*, *i* ∈ [1 … 4] [see Fig. 2(c)] can be found by solving the following relation for *l*_*P*_1__ and *l*_*P*_2__ based on the manipulator curvature *κ*_CC_, polar angle *ϕ*_CC_, and backbone length *l_M_* as follows:

(4) $$\phi_{\text{CC}}=\tan^{-1}\left(\frac{l_{P_{2}}-l_{M}}{l_{P_{1}}-l_{M}}\right),\;\kappa_{\text{CC}}=\frac{l_{M}-l_{P_{1}}}{l_{M}r_{P}\cos\left(\phi_{P_{0}}\right)}$$

where *ϕ*_*P*_0__ is the polar angle of the first tendon placement with respect to (w.r.t.) the local frame *x*-axis. The geometrical relations between the length of the opposing tendons for *l*_*P*_3__ and *l*_*P*_4__ are given as follows:

(5) $$l_{P_{3}}=l_{P_{1}}\left(\frac{1-\kappa_{y}r_{P}}{1+\kappa_{y}r_{P}}\right),l_{P_{4}}=l_{P_{2}}\left(\frac{1-\kappa_{x}r_{P}}{1+\kappa_{x}r_{P}}\right).$$

#### 2) Introducer Translation Model

As described in Section II-B4, the introducer helical spring acts as a ramp to linearly translate the coil attachment point to the valve introducer. The valve introducer translation is equal to the spring pitch at the contact point, which is a function of the manipulator bending angle *θ* (in radian). The coil attachment is fixed by the slider constraint at 45° counterclockwise (CCW) with respect to its actuator. The length of the virtual line passing through the coil attachment (*l_C_*) on the curved cylindrical shape is estimated as follows:

(6) $$l_{C}=l_{M}-\theta r_{S}\cos\phi_{I}$$

where *ϕ_I_* is the angle between the manipulator’s bending direction and the location of the coil attachment, and *r_S_* is the radius of the coil spring, which is equal to 10.8 mm. By knowing the rotational angle (*ϕ_a_*) of the introducer actuator, the introducer length (*l_I_*) can be calculated as follows:

(7) $$l_{I}=\frac{\phi_{a}}{360}\frac{l_{C}}{l_{M}}.\;\text{pitch}\;$$

where *pitch* is equal to 12 mm in the straight configuration.

The distance between the manipulator tip and target site *l_I_* can be acquired by the surgical navigator estimation in the *Z*-direction (14). Therefore, (7) can be solved to find *ϕ_a_* and transferred to the controller for introducer actuation.

#### 3) System Mechanics: External, Body, and Tendon Force Effect

PVW is employed to account for the change in the system states *q* as a result of actions associated with body load (here manipulator *w_C_M__* and introducer *w_C_I__* weight), external load (point *w_F_* and distributed *w_σ_* type), tendon tension *w_P_*, and beam bending stiffness *w_K_*. As a result, the robot maintains a CC shape with modified states to accommodate the effect of such loads. In the rest of this article, we use *X*_,*q*_ = ∂*X*/∂*q* to represent partial derivatives, where *X* is a dummy variable.

The action for an external point load at any location can be derived as *w_F_,q__* = *f_F_* · *ρ*_*F*_,*q*__, where *f_F_* = [*f_F_x__*, *f_F_y__*, *f_F_z__*] is the exerted point force and *ρ_F_* is the exertion point location vector, both expressed in the global frame. *ρ_F_* is calculated based on (1) and by substituting *θ_F_* into *θ* for forces exerted at a location with bending angle *θ_F_* along the robot manipulator or by substituting *l_F_* into *l_I_* for forces exerted at a location on the introducer with distance *l_F_* from the manipulator tip [see Fig. 2(d)].

The action for uniformly distributed external load vector *σ* on the manipulator can be modeled as *w_σ_,*q*__* = *f_σ_* · *ρ_σ_,q__* based on an equivalent point external load vector *f_σ_* = *σ*Δ*θ_σ_*/*κ* applying at the geometric center of the arc range *Δ* *θ_σ_* = *θ*_*σ*_1__ − *θ*_*σ*_0__ on which the force is exerted [see Fig. 2(d) and Appendix A]

(8) $$\rho_{\sigma }={\left(T_{\phi }\cdot T_{\theta_{\sigma_{0}}}\cdot \frac{3}{2\pi \kappa }\left[\begin{array}{c} -\sin\left(\text{Δ}\theta_{\sigma }\right)\\ 0\\ \cos\left(\text{Δ}\theta_{\sigma }\right)-1\\ 1 \end{array}\right]\right)}_{1\ldots 3}.$$

Frictional forces are the most common type of external forces for catheters and flexible robots. Assuming a uniform external force *σ* radial to the manipulator curve, the equivalent load becomes

(9) $$f_{\sigma }=\frac{\sigma \text{Δ}\theta_{\sigma }}{\kappa }\cdot {\left(T_{\phi }\cdot T_{\left(\theta_{\sigma_{0}}+\frac{\text{Δ}\theta_{\sigma }}{2}\right)}\cdot \left[\begin{array}{c} 1\\ 0\\ mu\\ 1 \end{array}\right]\right)}_{1\ldots 3}$$

where *μ* is the coefficient of friction between the robot body and tissue.

Similarly, the manipulator weight, i.e., distributed body load due to gravity *σ_M_*, acts at the entire manipulator arc center of mass with *θ*_*σ*_*M*0__ = 0 and Δ *θ*_*σ*_*M*__ = *θ* as follows [see Fig. 2(d)]:

(10) $$\rho_{C_{M}}=\frac{3}{2\pi \kappa }\cdot \left[\begin{array}{c} -\sin(\theta )\cos(\phi )\\ -\sin(\theta )\sin(\phi )\\ \cos(\theta )-1 \end{array}\right]$$

and *f_σ_M__* = *m_M_ g*. Then, for the manipulator weight action, we have *w_C_M_,q___* = *m_M_ g* · *ρ_C_M_,q___*, where *m_M_* is the manipulator mass, and *g* = [0, 0, −9.81]^*T*^ is the gravity acceleration vector. The introducer weight is assumed to be exerted at the middle of these elements as *ρ_C_I__* = *ρ_T_* (*l_I_*/2) [see Fig. 2(d)]. Then, the introducer weight virtual work becomes *w_M_I_,q___* = *m_I_ g* · *ρ_C_I_,q___*, where *m_I_* is the introducer mass.

The effects of the tendons’ tension can be modeled based on 1) tendon tension, i.e., the tendon pairs’ pulling force *p_i_*, or 2) equivalent curved beam, i.e., the resultant change in the manipulator’s resting curvature *κ*_*x*|*y*_0__ due to the tendon active lengths *l_P_i__* and the increase in the manipulator structural bending stiffness *k*_*x*|*y*_ due to the tensioned tendons’ cross section.

The bending virtual work *w_P_* of the resultant momentum *τ_P_* = [*τ_x_*, *τ_y_*, 0]^*T*^ from the tendon tensions *p_i_* in the tangent frame is given as follows:

(11) $$w_{P,q}=\left(\tau_{x}\kappa_{x,q}+\tau_{y}\kappa_{y,q}\right)l_{M}$$

where *τ_x_* = (*p*_3_ − *p*_2_ − *p_μ_x__*)*r_P_* is the moment of tendons placed along the *y*-axis (tendons 2 and 3). Similarly, *τ_y_* = (*p*_1_ − *p*_4_ − *p_μ_y__*)*r_P_* is the moment of tendons placed along the *x*-axis (tendons 1 and 4). *r_P_* is the tendon route offset from the manipulator center, and *p*_*μ*_*x*|*y*__ is the tendon tension due to internal friction forces that causes a moment around the local *x*|*y* axes.

Alternatively, the bending virtual work of the equivalent curved beam *w*_*K*_,*q*__, as a result of changing the tendon active lengths, can be found as follows:

(12) $$w_{K,q}=\left(k_{x}\kappa_{x,q}\left(\kappa_{x_{0}}-\kappa_{x}\right)+k_{y}\kappa_{y,q}\left(\kappa_{y_{0}}-\kappa_{y}\right)\right)l_{M}$$

where $\left[k_{x},k_{y},k_{z}\right]=\left[\frac{E}{4},\frac{E}{4},\frac{G}{2}\right]\pi (r_{M_{2}}^{4}-r_{M_{1}}^{4})$ is the equivalent beam bending and torsional stiffness, *r*_*M*_1|2__ is the manipulator inner–outer radius, and *κ*_*x*|*y*_ and *κ*_*x*_0_|*y*_0__ are found as in (4) (tendon CC relation).

The method in (11) relies on the measurement of the tendons’ tension, which is prone to noise and poses technical challenges in implementing tension sensors. On the other hand, the method in (12) is a function of the tendon geometry (i.e., cross-section area and offset from the manipulator axis) and active lengths, which can be easily measured based on the encoder readings from the pulling mechanism motors. We use the latter method due to its robustness and ease of measurement for our forward models. However, the virtual work from both methods is theoretically equal, i.e.

(13) $$w_{K,q}=w_{P,q}.$$

We use this equality to identify external forces, unmodeled internal frictional forces, and for our hybrid force/position control frameworks.

#### 4) System Forward and Inverse Mechanics

The system total virtual work is reported as follows:

(14) $${\bar{w}}_{,q}=w_{C_{M,q}}+w_{C_{I,q}}+w_{F_{,q}}+w_{\sigma_{,q}}+w_{K_{,q}}$$

where, in theory, we can substitute *w_K_* with *w_P_*. The FM and IM maps of the manipulator are based on the system balance of virtual works as ${\bar{w}}_{,q}=0$. The abovementioned relation results in a system of nonlinear equations (three relations for the inverse and two for the forward case) with the following set of variables [*κ_x_*, *κ_y_*, *l_I_*, *κ*_*x*0_, *κ*_*y*0_, *f_F_x__*, *f_F_y__*, *f_F_z__*, *f_σ_x__*, *f_σ_y__*, *f_σ_z__*]. Note that we assumed that the location of the point external load (*θ_F_* or *l_F_*) and the range on which the distributed external load is applied (Δ*θ_σ_*) are known. These are known based on observing the contact location in the robot cameras and estimating the distributed load range based on the inserted length of the robot, i.e., slider motion feedback, into the patient body.

In an FM framework based on (14), *l_I_* is part of the inputs and is fixed (already set). Hence, the system balance of virtual works should be derived w.r.t. *q*_FM_ = [*κ_x_*, *κ_y_*], instead of *q* = [*κ_x_*, *κ_y_*, *l_I_*], as ${\bar{w}}_{,\kappa_{xy}}=0$. A convex optimization problem can be formulated for any set of the abovementioned variables (three for the inverse and two for forward maps) to satisfy the relation for ${\bar{w}}_{,q}({\bar{w}}_{,\kappa_{xy}})$ and formulate forward/inverse maps for our continuum robot.

The FM for shape observation and a hybrid shape control and force observer framework are discussed in Appendix B. The remainder of this section discusses our newly developed theoretical frameworks for 1) hybrid force/shape observation by combining the information about tendon displacement and tension, and 2) hybrid force/position control based on tendon kinematics (displacement) instead of forces for robustness against tension sensing noises.

#### 5) Hybrid Force/Shape Observation

Force and shape observers (FSO) can be formulated similarly to force and trajectory controllers. In an IM formulation based on *w*_,*q*_ for force observation, the three [*f_F_x__*, *f_F_y__*, *f_F_z__*] or [*f_σ_x__*, *f_σ_y__*, *f_σ_z__*] variables are unknown, whereas [*κ_x_*, *κ_y_*, *l_I_*] are known based on the known robot’s tip trajectory *ρ_T_*, as in (2) and (3), and [*κ*_*x*0_, *κ*_*y*0_] from the known system inputs *l_P_i__*, as in (4). The nonlinear solution can be sought based on ${\bar{w}}_{,\kappa_{xy}}$ and initial guesses of [0, 0, 0] for the force values.

The extra information from the tendon tension sensors can be used to formulate a hybrid FSO. In other words, another set of two relations can be found based on the following equality:

(15) $$w_{KP,q_{\text{FM}}}=w_{K,q_{\text{FM}}}-w_{P,q_{\text{FM}}}=0.$$

By combining the abovementioned relation with *w*_,*q*_ in (14) we can formulate a convex optimization problem for five unknown variables, including [*κ_x_*, *κ_y_*]. The other three unknown variables can be any of the following sets:

1) [*p_μ_x__*, *p_μ_y__*], forming a redundant optimization problem, for internal frictional force estimation when no external force is being applied (by default),
2) [*f_σ_x__*, *f_σ_y__*, *f_σ_z__*] for distributed frictional and contact load estimation (during the robot’s automated insertion), or
3) [*f_F_x__*, *f_F_y__*, *f_F_z__*] for point load estimation, e.g., tip load during heart valve placement (when a contact is detected via observing a sudden jump in the tendon tension measurement values).

The optimization problem is solved numerically based on initial guesses [*κ*_*x*_CC__, *κ*_*y*_CC__] and [0, 0] or [0, 0, 0] for the unknown tendon or external forces.

The controller switches between these force observation modes. Upon switching to distributed or point external load observation, the values for [*p_μ_x__*, *p_μ_y__*] are set based on the most recent observed values.

### D Contact Aware Planning and Navigation

Safe navigation of the robot involves the following [see Fig. 2(a) and (d)]:

1) following a trajectory that converges to a target location *ρ_T_* (i.e., the heart valve),
2) passing always through the safe radius of *δ_E_* and a predefined insertion point *ρ_E_* on the patient body,
3) preventing excessive contact forces via real-time tendon tension observation,
4) stopping the insertion in the case of observing large contact force.

The proposed method benefits from minimal initial positioning requirements for the robot manipulator base *ρ_B_* and information about the precise location of the patient’s heart valve *ρ_T_*. As a result, the operator has only to roughly align the robot base with the insertion and heart valve locations only. Then, the control method automatically performs safe insertion via the robot base sliding displacement *l_S_* and manipulator bending inputs *l_P_i__* or [*κ*_*x*0_, *κ*_*y*0_]. The bending is commanded by the surgeon guiding the manipulator with a haptic interface (Novint Falcon, USA) while keeping a button on its handle. Its directional input is used by the controller to identify the cables to be shortened or released. The controller keeps the releasing cables’ tension at a set value by using the PID closed-loop control (settling time around 2 s). This set value is small enough (0.3 N) to prevent them from becoming loose or reversing the manipulator. This strategy keeps the manipulator dexterous enough to reverse in every position with a minimum delay. The motion continues until the manipulator tip *ρ_M_* aligns with the heart valve target location *ρ_T_* from where the introducer is utilized for reaching the target.

The robot is suspended on a 6-DOF robotic arm with the manipulator axis initially toward the $-{\hat{z}}_{0}$-axis (i.e., perpendicular to the patient bed surface). The operator may manually move the robotic arm to position it with the introducer tip slightly lifted away from the patient body and roughly in the same plane that contains the manipulator axis, the insertion, and heart valve commissure points. In a realistic scenario, this motion plane (*xyz*)_*R*_ is angled $\phi \approx \tan^{-1}\left(\frac{70}{90}\right)\approx 37.9^{\circ }$ around the ${\hat{z}}_{0}$-axis of the system global frame (*xyz*)_0_ with ${\hat{x}}_{0}$-axis along the patient body height [see Fig. 2(a) and (e)].

During the insertion, we assume that the robot is initially positioned with the introducer tip just touching the patient’s body and the insertion point remains on the manipulator backbone. Therefore, to find the desired manipulator curvature *κ* that guarantees the robot’s passage through the insertion point, the insertion point position vector during the insertion *ρ_BE_* = [*x_BE_*, 0, *z_BE_*] w.r.t. the manipulator base frame (*xyz*)_*B*_ should maintain a distance equal to the curvature radius κ*^−1^ from the curve center at [κ*^−1^, 0, 0] w.r.t. the manipulator base fame (*xyz*)_*B*_, as shown in the following equation [see the side view in Fig. 2(a)]:

(16) $$\kappa^{{*}^{-2}}={\left(\kappa^{{*}^{-1}}-x_{BE}\right)}^{2}+z_{BE}^{2}\to \kappa^{*}=2x_{BE}/\left(x_{BE}^{2}+z_{BE}^{2}\right)$$

where *x_BE_* is found based on the initial position of the robot set by the operator and *z_BE_* = *l_M_* + *l_I_* − *l_S_*.

The robot’s inverse hybrid shape control and force observation are employed during the insertion task, i.e., *l_P_i__* = FSO(κ*) as described in Section II-C4 and Appendix B-B. When an excessive force value *f*_*σ*_max__ is observed, the tension *p*_*i*_max__ in the closest tendons to the force contact location, with indices *i_σ_*, is recorded and considered as a threshold to limit their tension *p_i_σ__* = *P*_*i*_max__. This limits the exerted force by the robot toward tendons *i_σ_*. The closest tendons are the ones that satisfy the following relation:

(17) $$\begin{array}{c} \left(-{\left(T_{\phi }\cdot T_{\theta_{\sigma }}\right)}_{1\ldots 3\times 1\ldots 3}^{\text{T}}\cdot f_{\sigma_{\max}}/\left\|f_{\sigma_{\max}}\right\|\right)\\ \cdot {\left[\cos(\phi_{P_{i}}),\sin(\phi_{P_{i}}),0\right]}^{\text{T}}>0 \end{array}$$

where *ϕ_P_i__* ∈ {0, *π*/2, *π*, 3*π*/2} is the placement polar angle for the *i*th tendon, ‖*x*‖ is the 2nd norm (i.e., vector length) operator, and *θ_σ_* = *κ*(*l_M_* − *l_S_*/2) is the bending angle of the location along the manipulator backbone on which the equivalent distributed external load is exerted.

Such a strategy prevents excessive force, but it may result in a small deviation in the robot insertion point *ρ_BE_*. We may use the capped tension values along with (11), (13), and (14) to estimate the robot insertion point *ρ*_*BE*_*ϵ*__ via the robot FK. The deviated insertion point should remain at a safe distance *ϵ* from the initial insertion point, i.e. ‖*ρ_BE_* − *ρ_BE_ϵ__*‖ < *ϵ*, unless the procedure is terminated for safety concerns, i.e., when maintaining both the insertion point and tissue force safety is not possible.

The presented automation framework is robust against the manipulator and limited patient movements during the procedure, as long as the manipulator base remains perpendicular to the patient’s bed and the relative position vector of the insertion point w.r.t. the manipulator base *ρ_BE_* is known.

The alignment of the introducer tip with the heart valve location *ρ_T_* marks the end of the robot’s safe navigation stage. This alignment can be detected either by the operator via observing the heart valve in the introducer tip or when the value of *κ* found from both (2) (based on the patient’s heart valve position, *κ_T_*) and (16) (during the automated insertion, κ*) becomes equal to κ* = *κ_T_*. The latter requires the aortic root pose in the global frame as measured by a digitizer probe, as shown in controller Fig. 5.

Then, the control law during the automated insertion, i.e., while κ* < *κ_T_*, maintains a safe insertion route and keeps safe interaction force values based on the following tendon tension regulation rule:

(18) $$\{\begin{array}{cc} l_{P_{i}}=\text{FSO}\left(\kappa^{*}\right) & f_{\sigma }<f_{\sigma_{\max}}\\ l_{P_{i}}\;\text{and}\;p_{i_{\sigma }}=p_{i_{\max}} & f_{\sigma }>f_{\sigma_{\max}}\;\text{and}\;\|\rho_{BE}-\rho_{BE_{\epsilon }\|<\epsilon }\\ \;\text{Terminate!}\; & \;\text{otherwise}\; \end{array}.$$

### E Surgical Navigator

The camera images were captured and processed using a navigation software developed in C++, which integrated the FISBA camera API and the OpenCV libraries. One camera was taken as a reference, whereas the other two were rotated +60° and −60° with respect to the reference camera. Each camera was calibrated to obtain intrinsic parameters that model the image formation process, as well as extrinsic parameters that provided the relative poses between the three cameras. The calibration process and the key functionalities of the surgical navigator were extensively presented in [28] and [33]. Specifically, the software was able to realign the horizon between the cameras, stitch the three views together, and simulate an augmented reality view of the valve positioning [33]. Additionally, the software could compute and show the 3-D coordinates of the user-selected points if they were visible in at least two camera views. For instance, by selecting one point on the anatomy and one point on the valve, the surgeons were able to find the error in depth and manually move the valve [28].

This 3-D point computation was further utilized to be integrated as feedback into robot control algorithms for autonomous alignment based on anatomical landmarks. Given that, the landmarks are determined in the camera reference system, whereas the control algorithms need them in the robot reference system. Thereafter, an additional calibration procedure was required to get the transformation matrix.

**Fig. 4:**
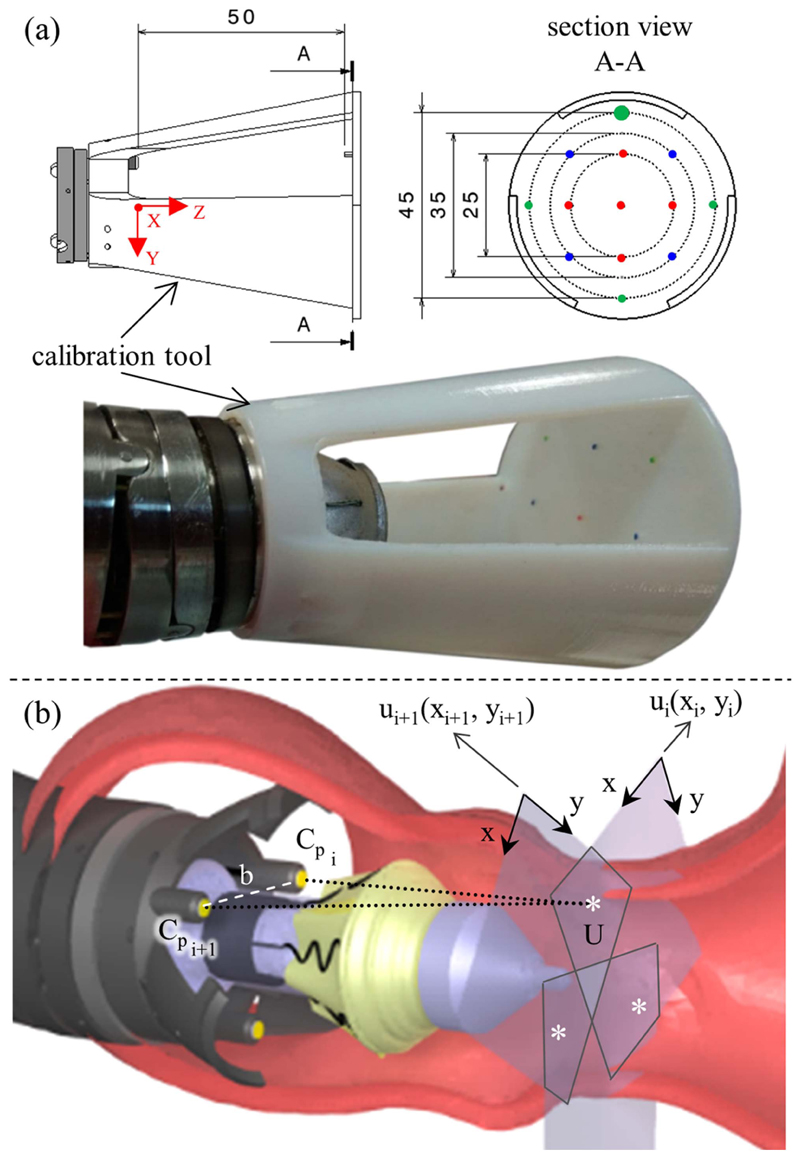
Surgical navigator developed thanks to the integrated tip cameras. (a) Calibration tool with the reference points marked with different colors to facilitate their identification in the camera views. (b) Simplified problem associated with the triangulation of the 3-D *U* point, based on the knowledge of its projection on the camera image planes, *u_i_* and *u*_*i*+1_.

This calibration has been determined by attaching a calibration tool that was attached to the distal end of the manipulator [see Fig. 4(a)]. The calibration tool allows for relating the camera reference systems {C_V_} to the reference system of the surgical robot, {M}, through the identification of its reference points, known in {M} and named *P^M^*. The reference points can also be identified in {C_V_}, as detailed in Fig. 4(b), providing *P^C_V_^*.

The 3-D position of the points [*U_x_* *U_y_* *U_z_*]^*T*^ was computed through stereo-triangulation between couples of images, according to (19)–(21), starting from their 2-D projections (*u_X_i__*, *u_Y_i__*) and (*u*_*X*_*i*+1__, *u*_*Y*_*i*+1__). The triangulation equations were simplified by considering the image pair after rectification, which aligned corresponding points on the same line in both images

(19) $$U_{Z}=\frac{b*f_{x}}{u_{X_{i+1}}-u_{X_{i}}}$$

(20) $$U_{X}=\frac{u_{X_{i+1}}*U_{Z}}{f_{x}}$$

(21) $$U_{Y}=\frac{u_{Y_{i+1}}*U_{Z}}{f_{y}}$$

where *b* is the stereo camera baseline in mm and *f* corresponds to the focal length of the cameras.

All the pairs of corresponding 3-D points *P^C_V_^* and *P^M^* were used to estimate the calibration transformation relating the reference systems, $T_{C_{V}}^{M}\left(R_{C_{V}}^{M},t_{C_{V}}^{M}\right)$ with a closed-form least-squares method that minimizes the following:

(22) $$\frac{1}{N}{\sum_{n=1}^{N}\left|R_{C_{V}}^{M}P_{n}^{C_{V}}t_{C_{V}}^{M}-P_{n}^{M}\right|}^{2}.$$

We adopted *N* = 13 matching points and computed $T_{C}^{M}=\left\{R_{C_{V}}^{\text{M}}|t_{C_{V}}^{M}\right\}$ from singular value decomposition (23)

(23) $$\begin{array}{c} {\left(P^{C_{V}}P^{M}\right)}^{\prime }\overset{\text{SVD}}{\Leftrightarrow }USV^{\prime },\\ R_{C_{V}}^{M}=VU^{\prime },\\ t_{C_{V}}^{M}=\frac{R_{C_{V}}^{M}}{N}\sum_{k=1}^{N}P_{k}^{C_{V}}+\frac{1}{N}\sum_{n=1}^{N}P_{n}^{M}. \end{array}$$

Once the calibration matrix $T_{C}^{M}$ is known, the software can provide the 3-D coordinates of generic user-selected points in the robot reference system as long as they are detectable in at least two camera views and vice versa.

During the operation, the surgeon can use the 3-D coordinates on the aortic commissures to determine the optimal valve pose in terms of position and orientation. When all three commissure points are selected, the software derives the circle passing through them and outputs the 3-D location of the circle center. This information is passed to the controller to center the manipulator and achieve the introducer’s autonomous positioning.

The theoretical tracking error of a 3-D point can be calculated as follows [34], [35]:

(24) $$\begin{array}{l} {\text{Δ}}_{X}=\frac{2*U_{Z}*\tan\frac{{\text{FOV}}_{H}}{2}}{{\text{C}}_{H}}{\text{Δ}}_{d},\\ {\text{Δ}}_{Y}=\frac{2*U_{Z}*\tan\frac{{\text{FOV}}_{V}}{2}}{{\text{C}}_{V}}{\text{Δ}}_{d},\\ {\text{Δ}}_{Z}=\frac{U_{Z}^{2}}{\text{b}*\text{f}}{\text{Δ}}_{d} \end{array}$$

where FOV_*H*_ and FOV_*V*_ are the horizontal and vertical fields of view, respectively. *f* is the reference camera focal length in pixels, and Δ_*d*_ is the matching error in pixels that depends on the user’s precision in selecting corresponding points in the two camera images. In our system, this accuracy value ranges from 0.25 mm to a maximum of some millimeters, when the Δ_*d*_ error becomes relevant.

### F Control and Automation Architecture

The controller is planted on the graphic user interface (GUI) of LabVIEW (NI, USA) running on a personal computer (Intel Core i7 CPU at 2.6 GHz) to actuate the servomotors by RS232 communications and enable real-time monitoring of the cable tensions. The haptic interface unit is connected via a USB interface to the GUI, providing 3-DoFs to the robot. The interface can command manual bending when the handle moves right–left (*x*-axis) or forward–backward (*y*-axis) and can generate force feedback on both axes. This haptic interface has included complex libraries to turn each motor and generate an equivalent force in any direction. The software and third-party haptic visualization packages (Force Dimension, Switzerland) were utilized for connecting the interface to the GUI. The motors are updated at 1000 Hz, which gives a smooth sense of touch based on the force observer described in Section II-C5.

**Fig. 5:**
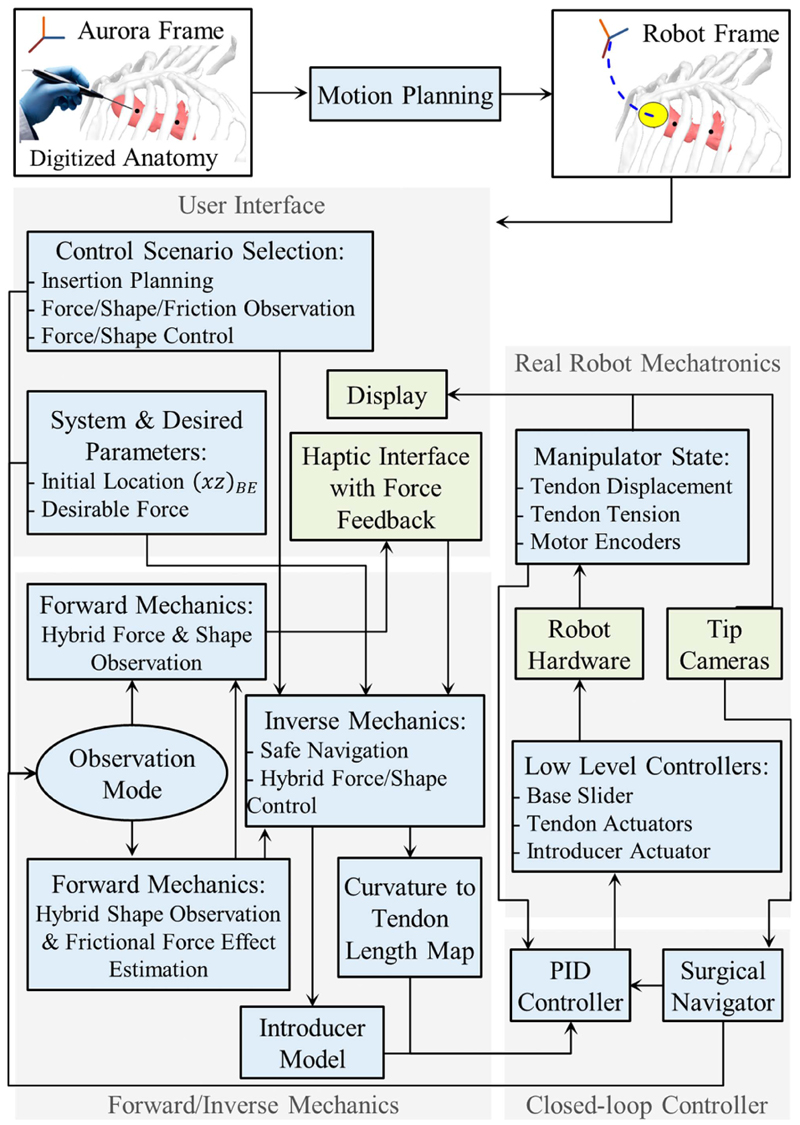
Control and automation system architecture. Blue boxes represent the software part of the system, and green boxes are the hardware part.

Even though the Falcon Novint is a relatively cheap solution, the performance of the device can be optimized by implementing dynamic modeling of the handle [36]. The LabVIEW GUI continuously transfers and receives data with MATLAB R2021 (Mathworks, USA) over the TCP/IP protocol in which the FK and IK models are planted. Moreover, the surgical navigator also sends its output to the GUI to achieve a unified controller (see Fig. 5). The real-time operation is assured by adjusting timing functions to precisely control the execution of loops in the program.

An Aurora (NDI Medical, Canada) electromagnetic tracking system (ETS) is utilized at the beginning of the surgical procedure to have an estimation of target release pose and robot base point necessary for motion planning.

## III Simulation and Experimental Studies

### A Insertion Motion Planning Performance

**Fig. 6:**
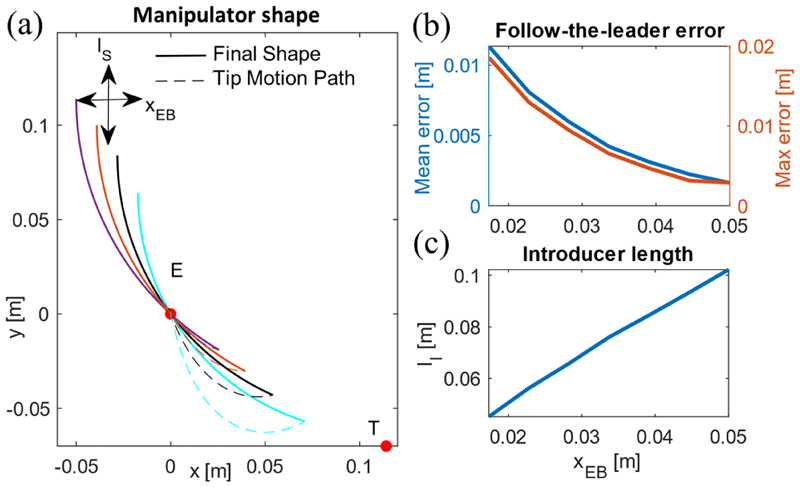
(a) Comparison of the final manipulator shape (solid line) and its tip motion path (dashed line) for different horizontal distances *x_BE_* of the manipulator base from the insertion point *E*. The motion stops when the manipulator tip aligns with the target position *T*. (b) Required introducer length *l_I_* to reach the target point and the mean and maximum follow-the-leader error values versus *x_BE_*. (c) Larger the distance *x_BE_*, the lower the follow-the-leader error but larger required introducer length. The manipulator *z*-axis positive direction is upward.

We presented a simple yet robust automation framework for the robot’s motion through an insertion site on the patient’s body and reaching the heart valve location in Section II-F. Fig. 6(a) presents the simulation results for the motion planning method performance versus different values of the manipulator’s base distance to the insertion site on the patient’s chest. We observed that a larger value for *x_BE_* [see Fig. 2(a)] results in a robot motion closer to a follow-the-leader case, i.e., a smaller mean error between the robot final shape and the robot tip insertion path [see Fig. 6(b)]. Such a motion suggests a smaller undesirable lateral deflection of the pathway during the insertion. However, this limits the manipulator penetration length and requires a longer motion range for the tip introducer [see Fig. 6(c)].

### B Experiment Scenarios and Procedures

**Fig. 7:**
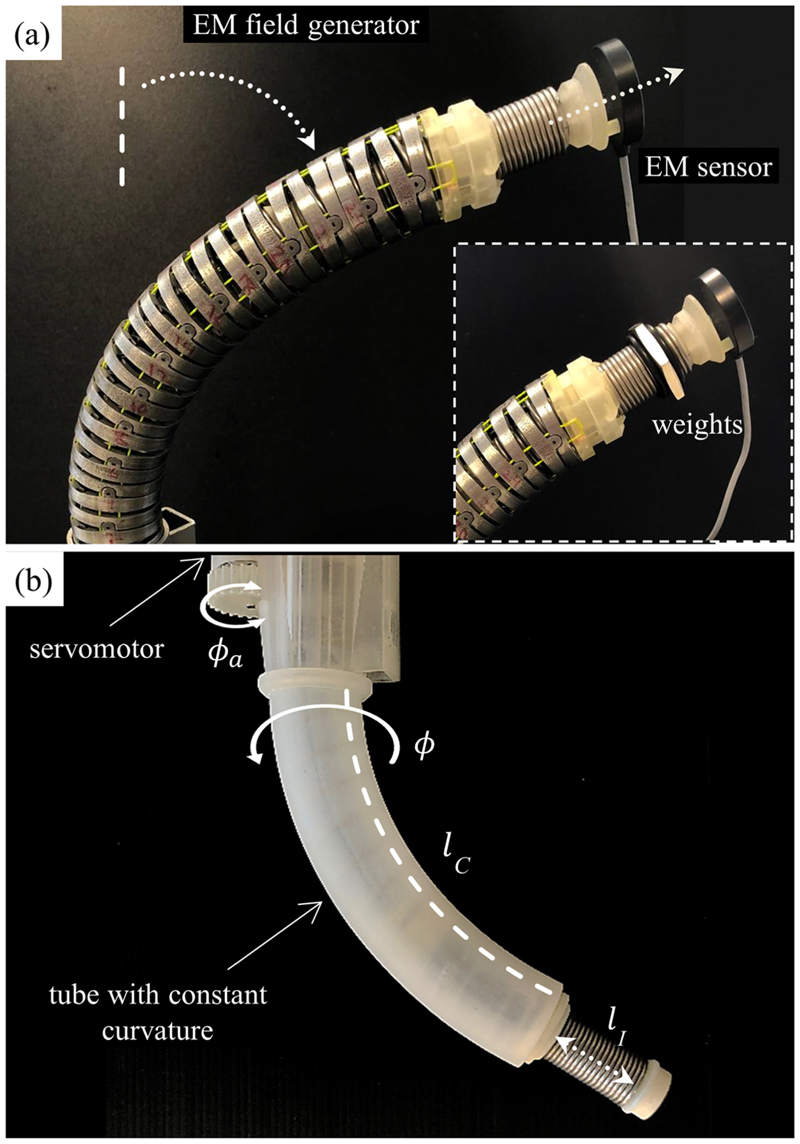
Experiments with simplified prototypes to validate the system. (a) FK and IK validation setup (inset: weights added to the introducer body). (b) Setup for validating the introducer translational model while the tube mock-up can rotate and provide various coil attachment lengths.

Initially, we performed several experiments to validate the new model that is described in Section II-C1 using a simplified manipulator without cameras [see Fig. 7(a)]. This change was motivated by the need to keep the delicate camera wires safe during the model adjustments. The introducer and its stiffness and weight on the manipulator were considered in the model.

Positioning performance was quantitatively assessed by tracking the introducer tip through the Aurora ETS. To synchronize the tracking system and the robot, both the aurora and the robot controller were implemented in LabVIEW.

#### 1) FM and IM Validation

**Fig. 8:**
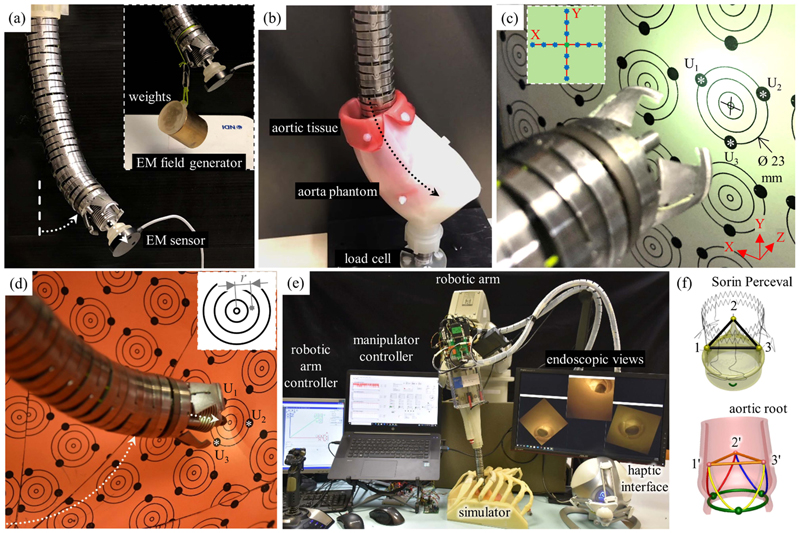
Experiments to validate the system. (a) FK, IK, and FSO validation setup; inset: force observer validation by four weights hanging from the manipulator tip. (b) Setup for validating forces on the body of the manipulator during insertion. (c) Setup for measuring the accuracy of the 3-D center estimation while the target board was repositioned by the red arrows. (d) Setup for validating the controller’s accuracy in reaching a target point. (e) Experimental setup for performing artificial valve delivery in a silicony mock-up. (f) Points in Sorin Perceval and commissure points in the aortic root that should match for an ideal positioning.

**Fig. 9:**
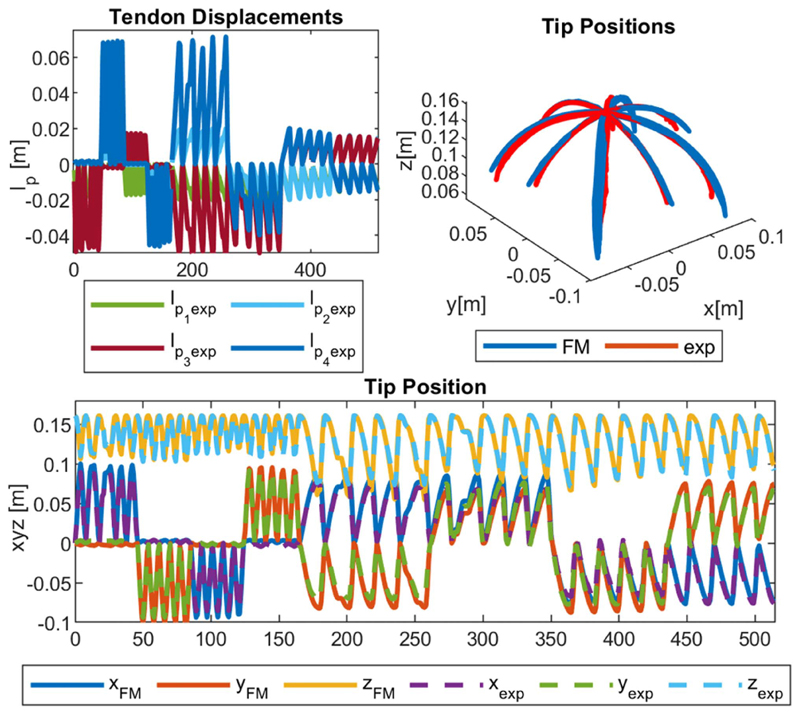
Results of the FM framework for the robot workspace (experimental cases 1).

**Fig. 10:**
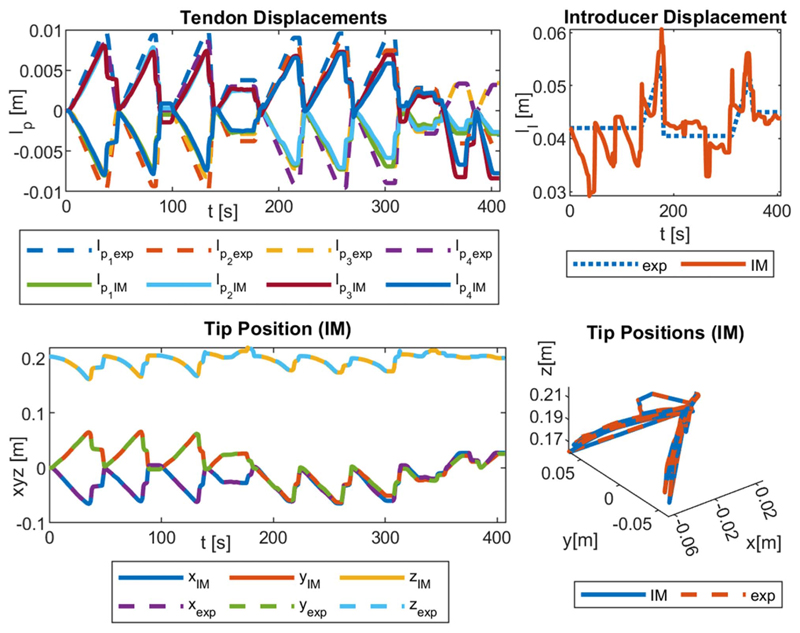
Results of the IM framework for experimental case 2. The accumulated error due to CC kinematics results in a large error in predicting the introducer linear motion.

**Fig. 11:**
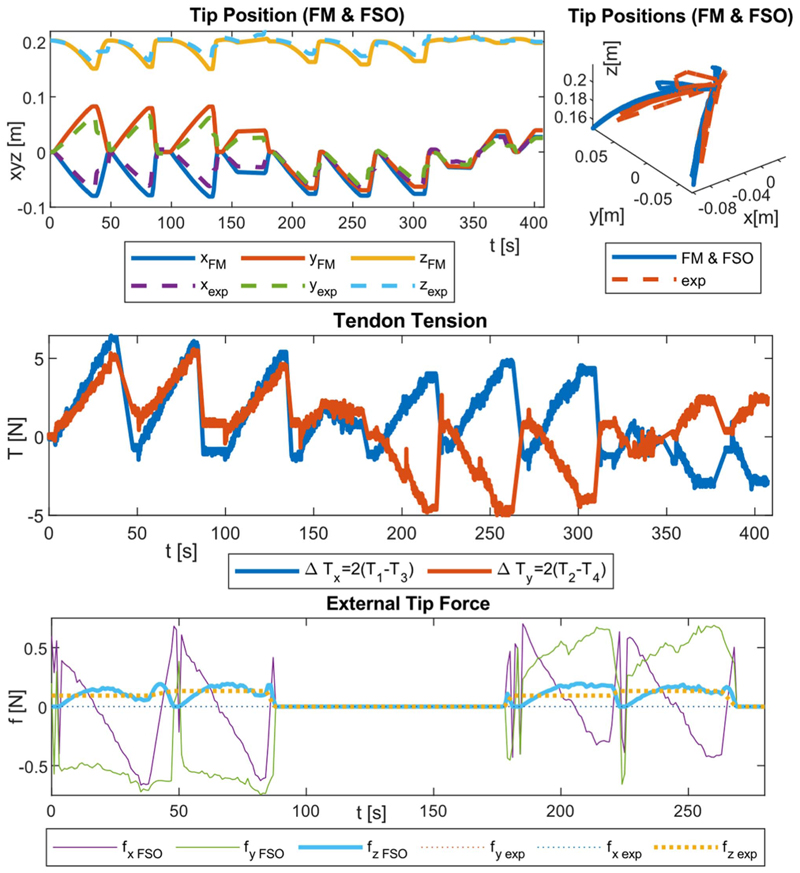
Results of the hybrid FSO framework for experimental case 2 with robot motion when external forces were applied. The force estimation results are relatively accurate in the *z*-axis direction. The accumulated error due to CC kinematics and the high stiffness of the system result in predicting unrealistically large lateral forces in the *x* − and *y*-axis directions.

**Fig. 12:**
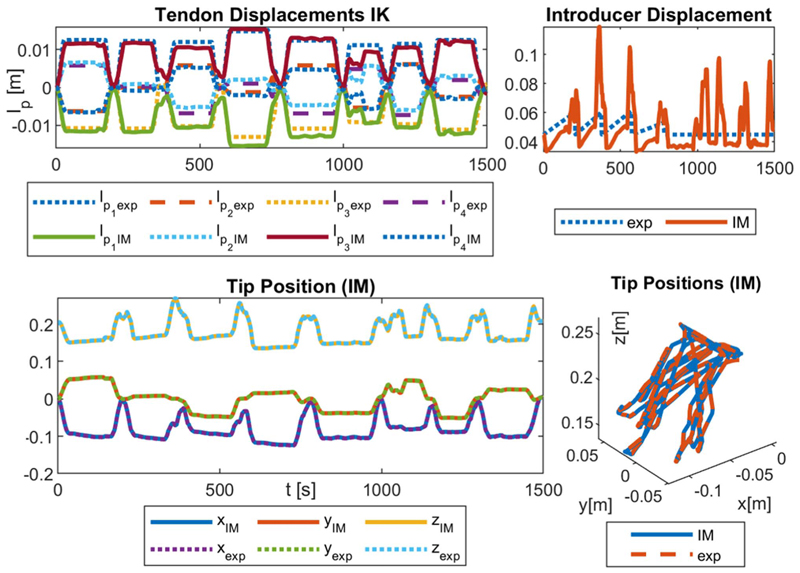
Results of the IM framework for experimental case 3.

**Fig. 13:**
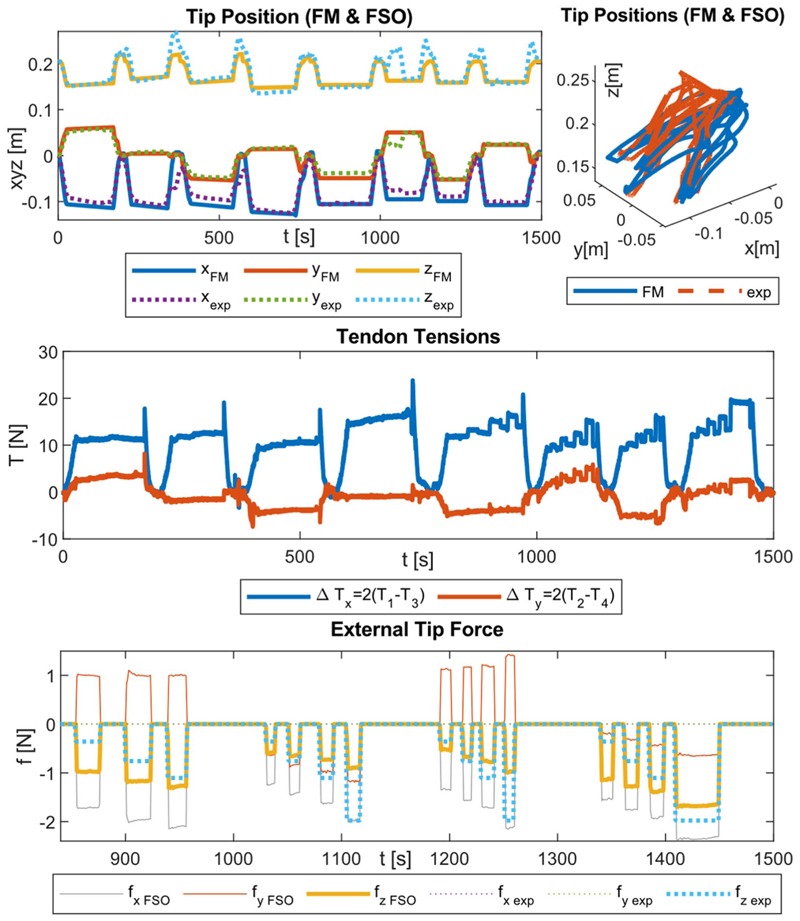
Results of the FSO framework for experimental cases 3 with robot stationery when external forces were applied.

**Fig. 14:**
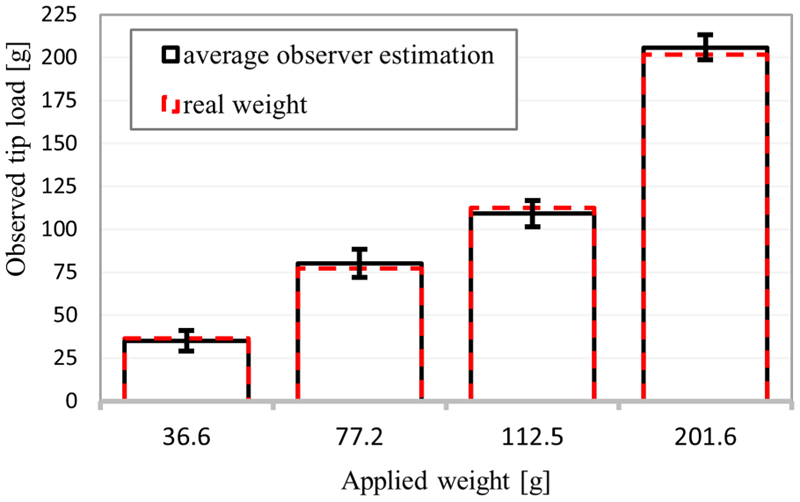
Force observer estimation after tendon tension release. Four different weights were applied to the robot tip.

Three experiment sets were carried out to verify the numerical performance of the 1) FK, 2) IK, and 3) hybrid FSO observation frameworks [see Figs. 7(a) and 8(a)]. Initially, 3-point moving average blocks filtered the cables’ tension measurements to eliminate the fluctuations on the sensors’ readout. In experiment set 1, the manipulator was laterally actuated to approach the workspace boundaries to verify our FK framework. The result is presented in Fig. 9. In case 2, the manipulator and introducer were actuated in four instances while carrying two weights (9.5 and 13.5 g) added to the introducer body to verify the presented IM and FSO frameworks in the presence of known external loads. The result is presented in Figs. 10 and 11. In case 3, the manipulator was bent to reach an arbitrary pose and then kept stationary. Different sets of weights (36.6, 77.2, 112.5, and 201.6 g) were added and removed from the system in different bent and extended configurations to investigate the presented IK and FSO performance in the presence of instantaneous external load disturbances. This experiment was repeated five times for a total of 20 measurements after releasing the residual cables’ tension. The result is presented in Figs. 12 and 13. In the latter case, an instance of an external force disturbance could be identified based on a sudden jump observable in the tendon tension values (see Part II of the Supplementary Material). The manipulator tendon tension measurements during the unloaded configurations were used to estimate the effects of the system internal friction (*p_μ_*). Then, the identified frictional effects and the tendon tension measurements during the loaded cases were used to estimate the added weight to the robot tip. The result is presented in Fig. 14. The tip displacements during these experiments were recorded to assess the shape and force observation approaches.

The accuracy of the model was verified in 515, 408, and 1500 sampled points across the experimental cases 1–3, respectively. The error was calculated based on the Euclidian distance between the simulation and experimental results for the robot tip in mm (absolute error) and the relative error with respect to the manipulator length reported in percentage (see Table I). As a measure of the potential real-time performance of the proposed framework, the calculation frequency is reported in hertz as the number of unique experimental datapoints that are simulated in a second.

#### 2) Introducer Sliding Model Validation

The introducer translation model was checked using two curved 3-D-printed tubes. The tube curvatures have been acquired by the CAD software (SOLIDWORKS, Dassault Systems, France) by considering *θ* = 39° and 78°. We prototyped a simple introducer mechanism, and these tubes were positioned instead of the cable-driven manipulator. The tubes guarantee a constant natural line length (*l_M_*) and bending angles (*θ*) while by rotating the tube around the normal base plane axis (*ϕ*), the coil attachment line (*l_C_*) can vary accordingly [see Fig. 7(b)]. The translation of the introducer (*l_I_*) was considered by actuating the motors at known angles (e.g., 360° and to 0° to simplify the procedure) and varying *ϕ* from 0° to 360° in steps of 60°. At each step, *l_I_* was measured using a digital caliper and compared with the values acquired by (7).

#### 3) Distributed External Force Estimation Due to Friction

The performance of the automatic insertion framework in the presence of distributed frictional forces on the body was evaluated in a set of in vitro experiments based on a custommade aortic phantom. The phantom is mounted on a 6-DoF force/torque sensor (Nano17, ATI Industrial Automation, USA). The phantom consisted of a plastic shell with an artificial aortic tissue (Thoracic Aorta, LifeLike BioTissue, Canada) covering its internal layer.

The coefficient of friction *μ* between the manipulator material, i.e., polished stainless steel, and the aortic tissue phantom was evaluated by pulling the force sensor surface on the tissue multiple times and recording the observed normal and tangent forces to the tissue surface.

**Fig. 15:**
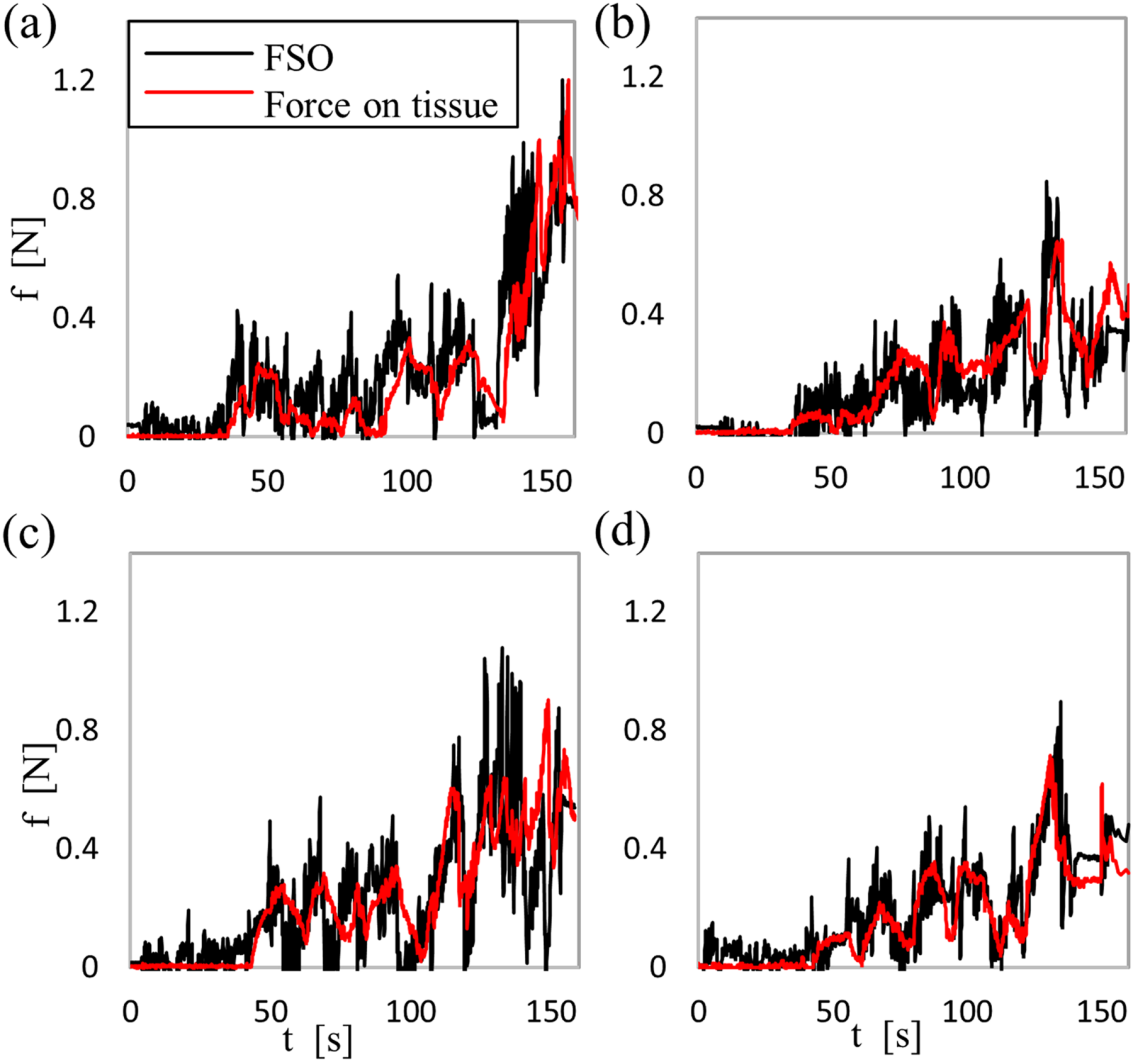
Insertion performance. (a) Manual insertion without safety feature. (b) Manual insertion with activated safety and force feedback. (c) Automated insertion without safety feature. (d) Automated insertion with activated safety and force feedback.

The robot was held straight on top of the phantom, and the manipulator was inserted through the phantom top opening while touching the convex side of the lumen [see Fig. 8(b)]. The distributed force range in (9) can be estimated based on the inserted length of the robot in the phantom Δ *θ_σ_* = *κ*(*l_M_* − *l_E_*). This experiment was repeated five times. The observed distributed lateral force values *f_σ_xy__* based on the presented FSO framework were compared with the force sensor data in the *xy*-plane (i.e., normal to the phantom surface) and presented in Fig. 15.

#### 4) Automatic Insertion Validation

The insertion planning was evaluated by a custom-made aortic phantom described in Section III-B3. The robot was held straight on top of the phantom [see Fig. 8(b)]. Four experimental cases were evaluated based on automated and manual insertion with force feedback safety criteria (via safety tendon tension threshold, *p*_*i*_max__) being activated or deactivated as described in Section II-D. In the cases of automated insertion, the manipulator curvature and corresponding cable shortening were calculated based on the phantom curvature. In the cases of safety force feedback, *p*_*i*_max__ was equal to 1 N and haptic device was activated.

#### 5) Visual Center Point Evaluation

The accuracy of the 3-D center estimating method was verified by a target board in which three points at 120° shifted angles on the circles of 23 mm (representing a common annulus size) have been printed [see Fig. 8(c) and Part III of the Supplementary Material]. Initially, one circle on the printed board was positioned at the origin of the imaging system, 3 cm distance from the camera plane. Then, three points were selected by the user in the camera videos, and the circle passing through these points was calculated. The calculated center point and diameter were compared with the printed ones (i.e., 23 mm in diameter). Furthermore, the target board was repositioned by 3 mm in the *X*- and *Y*-direction (i.e., from −3 to 3 mm by steps of 1 mm). The estimated centers were compared with the movements to calculate the errors and finally divided by the distance to the origin. The same experiment was repeated with the board positioned at 4 and 5 cm distance from the camera plane, and *U_Z_* [see Fig. 8(c)] was verified too.

#### 6) Motion Tracking Evaluation

The presented control framework was experimentally evaluated in tracking 3-D paths in the shape of a planar circle and a curved square by means of only the manipulator bending. The controller was also evaluated in tracking a planar square by means of both manipulator and introducer motions. To produce a planar circle, 360 points representing a circle of 80 mm in diameter were fed to the IK controller. To produce a curved square with 90 mm side lengths, 72 points dividing the square at equal distances of 5 mm were considered. In a more complex motion, a planar square with 120 mm side lengths was produced in a plane 20 mm lower in the *Z*-direction than the planar circle. The error was calculated based on the Euclidian distance between the experimental Aurora ETS points and the ideal targets. The average absolute error (mm) and relative error (%) with respect to the manipulator length have been reported in the first row of Table II.

Moreover, the aforementioned paths were repeated with the help of the surgical navigator feedback in a closed-loop control. The points representing the planar paths were scaled and printed on a target board in front of the surgical navigator. On the other hand, for the curved square path, the manipulator’s workspace was acquired in CAD software. The umbrella-like workspace was sliced into eight pieces and unfolded in a 2-D plane. The points representing the square were scaled and printed on a 250 grams per square metre (gsm) orange paper. The workspace was prepared by carefully gluing the boundaries of these eight pieces. The user had to select each point in at least two views to provide feedback to the controller. The tip trajectory is presented in Fig. 15, and the error is reported in the second row of Table II. The system in closed-loop IK control was further checked while a 13.5 g mass (equal to the Sorin valve cartridge mass) was added to the tip. The system repeated the same paths, and the error is reported in the third row of Table II.

#### 7) Automated Positioning in the Robot Workspace

The system controller was further evaluated in the manipulator’s workspace mock-up, where several concentric circles (with decreasing diameters of 23, 16, 8, and 2 mm) and commissure points at 120° shifted angles were printed on the unfolded orange paper [see Fig. 8(d) and Part IV of the Supplementary Material]. The commissure points will be utilized by the navigator to position the robot appropriately. The straight manipulator was held in the center of the workspace, and it bent toward one of the circles. The positioning task was interrupted when commissure points became recognizable by the user in the camera views. The user had to select each commissure point in two views, and the circle passing these points was calculated. The center point was fed to the IK model to reposition the manipulator toward the center. The average *U_Z_* was converted to a rotational angle of the introducer to actuate it outward. Meanwhile, the manipulator could autonomously compensate for small deviations caused by the introducer motion. The valve cartridge on the tip of the introducer was also modified to keep a graphite stick and consequently leave a point when it touches the group of circles on the umbrella workspace. In this experiment, the accuracy of the system to reach a point was evaluated by measuring the distance between the graphite point and the circles’ center [i.e., *r* in Fig. 8(d)] by means of a digital caliber. This experiment was repeated for ten different circle groups in the whole workspace.

#### 8) In Vitro Experimental Investigations and User Study

Finally, the robot’s performance in an in vitro simulated surgical scenario was evaluated. To this purpose, a patient-specific physical simulator, including rib cage, aortic arch, ascending aorta, and aortic root, was prototyped in ABS and silicone [28] [see Fig. 8(e)]. The simulator was prototyped from the tomography dataset of a patient’s anatomy as a reference for guiding the robot’s introducer and valve deployment by the surgical navigator [37]. The valve delivery procedure discussed in Section II-A was performed to deliver the Sorin Perceval S into the silicone replica of the aorta (see Part V of the Supplementary Material).

The coordinates of the ascending aorta and the aortic root were acquired by the Aurora ETS 6-DoF probe and registered in the motion planning algorithms (see Fig. 5). The manipulator was localized on the top of the simulator by a robotic arm (Mitsubishi Melfa, RV-3SB, Japan) to reach the valve expansion point as described in Section III-A.

**Fig. 16:**
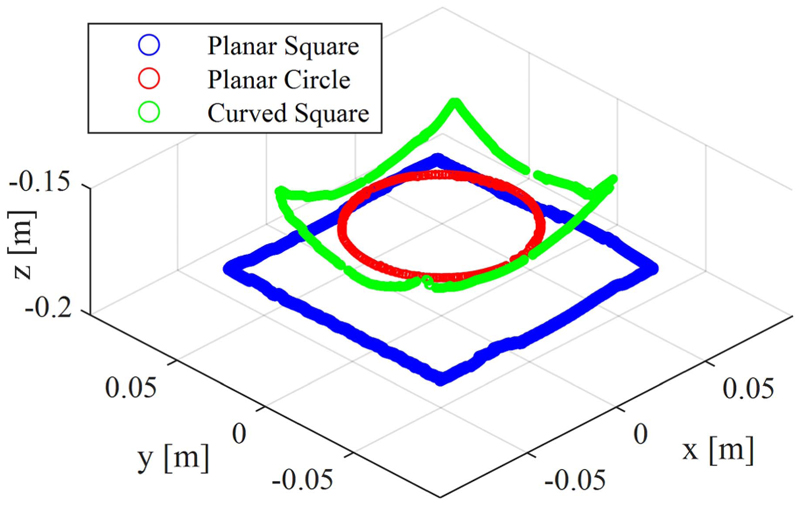
Trajectory tracking performance for three shapes: a planar circle, a curved square, and a planar square, based on closed-loop control frameworks. The manipulator *z*-axis positive direction is downward.

In this experiment, the arm pose was inserted into the kinematic model as the initial reference point (*S*). The user must choose the control scenario as insertion planning and force/shape observation. The user commanded the manipulator linearly inside the simulator from the second and third costal cartilage (point *E*) by exploiting the bending ability by the haptic interface [see Fig. 16(a)]. The commands for linear insertion were given by two buttons on the handle of haptic interface. The linear insertion (*l_S_*) was also counted by the motor encoder, and its translation transformation was multiplied in the FK model. Force feedback to the haptic interface helped the user avoid contacts with the surrounding simulator. A direct view of the anatomy to find the cutting entry in the beginning and the feedback of cameras while the manipulator was completely inside the simulator were helping to find the descending aorta and finally reaching the intervention site [see Fig. 16(b)]. The manipulator was stopped 3−4 cm behind the annulus while the tip was oriented to the aortic plane. By opening the flaps, the manipulator was anchored and the action of pushing the surrounding aorta avoided blocking cameras’ line of sight.

The user must select each commissure point in two views [see schematic illustration in Fig. 1(b) and real view in Fig. 16(c)]; then, the center point was fed to the IK and introducer model to drive the introducer to the aortic annulus autonomously [see Fig. 16(d)]. The user may update the commissure point selection during the introducer motion or in the later stage while the valve is placed in the aortic annulus. The user can manually rotate the valve to finely adjust the outflow frame. When all the requirements seem satisfied, the expansion stage can start with user commands in two stages to completely release the valve. The introducer will be retrieved inside the manipulator passing through the valve. After closing the flaps, the manipulator will be retracted from the simulator by combined bending and linear motions [see Fig. 16(e)].

The valve deployment accuracy was evaluated quantitatively after each release by measuring the misalignment between the Sorin Perceval S and the aortic root. To this purpose, after each delivery trial, the silicone aorta was carefully detached from the simulator, and the 3-D coordinates of commissure points on aorta and valves’ struts [see Fig. 8(f)] were acquired by the Aurora ETS and a digitizing probe (similar to the methods presented in [28]). The ideal position was acquired from surgeons’ indications and Sorin official instruction for use. The procedure was repeated ten times by the same user, starting with repositioning the robotic arm to recharge the valve cartridge and perform a new valve release. All points were processed to create the commissure and valve strut triangles [see Fig. 8(f)] to calculate delivery errors. The distance between two centroids (*d*) and intersecting angle of two planes (*α*) are reported in Table III. For calculating the rotational error, first, the valve triangle was projected onto the commissure plane, and then the average angle between each pair of three vertices (*β*) was considered. The results are reported in Table III.

## IV Results

The robotic system has been utilized in various experiments to check the controller performance individually and the valve delivery procedure at the end. The employed methodologies are described in Section III, with the theoretical modeling and control background illustrated in Section II.

### A FM and IM and Force/Shape Observer

Figs. 9–13 present the comparison between the simulation and the experimental results of FM, IM, and FSO in three experimental cases, as explained in Section III-B1. The results show the good accuracy of the FM and FSO simulations in predicting the experimental results. Moreover, the IM results show good numerical stability of the inverse problem and good accuracy of simulations in predicting tendon displacement inputs. The error analysis for the FM, IM, and FSO cases are reported in Table I.

The results in Fig. 9 show an average 6.3% (10 mm) error for FM, thus highlighting the reliability of the presented modeling framework based on CC assumptions. As briefly mentioned before, a more accurate model of the system based on variable curvature kinematics is developed by using the *TMTDyn* package [38], resulting in almost the same error values for the same set of experiments. This highlights the fact that the observed errors are due to manufacturing imperfections in the system rather than the kinematic assumption in the presented modeling framework. Hence, we proceeded with integrating a vision-based closed-loop controller to compensate for the errors instead of increasing the complexity of our theoretical modeling framework.

The robot tip trajectory was fed to the IM framework to calculate the required actuation inputs. These inputs were then used with the FM to calculate the resulting tip trajectory, which is used to calculate the numerical performance of the IM framework (see Figs. 10 and 11). The 0.2% (0.3 mm) error for the IM shows high numerical accuracy and stability of the proposed framework in solving the system inverse map. However, this is not necessarily equivalent to the feedforward control performance of the system due to the observed error in the FM case. This discrepancy can be seen based on the resulted values for the tendon *l*_*p*_*i*_IM_ and introducer *l*_*I* IM_ displacement compared to the actual experimental values (*l*_*p*_*i*_ exp_ and *l*_*I* exp_). Hence, a closed-loop control architecture with a PID error compensation term is used and tested as explained below.

We observed a similar 11.9% (18.9 mm) error in shape observation in the cases of IM and FSO despite the presence of instantaneous external force disturbances at the robot tip, which were unknown in the case of FSO (see Figs. 12 and 13).

The load estimation results were relatively accurate in predicting the force value in the exerted direction (i.e., global frame *z*-axis). However, the prediction of the overall force vector value and direction was not optimal by predicting large lateral force values (i.e., global frame *x*, *y*-axis), regardless of whether the robot was in motion (see Fig. 12) or in a stationary (see Fig. 13) state during the external force disturbance.

The FSO accuracy in the *Z*-axis was examined using four different weights {36.6, 77.2, 112.5, and 201.6 g} hanging on the tip. Then, the presented algorithm resulted in average values of {34.8±6.0, 79.9±8.2, 108.8±7.5, and 205.0±7.0 g} with respect to the mentioned weights, as shown in Fig. 14. The average pose deviation when applying a load was equal to 0.0072 mm/g.

This highlights the need for a careful tension release upon observing a sudden change in the tendon tension values (signaling the exertion of an external force) and before the load observation task, as detailed in Section II-C5. Furthermore, this shows the fact that it is tricky to rely on tendon tension measurements for control and observation tasks due to the highly noisy sensor data and influence of hard-to-model frictional effects in the system. On the other hand, a modeling framework that relies on tendon length measurement is not prone to such issues.

### B Introducer Sliding Performance

The introducer model has been validated in 12 configurations by 2 tube mock-ups and a 360° rotation of its actuator. This experiment has resulted in a 0.8±0.5 mm error in forward motion and a 1.3±0.7 mm error in backward motion.

### C Coefficient of Friction Estimation

Fig. 15(a) presents the comparison between the experimental and observed values for the distributed forces normal to a phantom surface during the insertion experiments, as explained in Section III-B3. The results show good agreement between the observed values and the real results, with an average 0.10±0.10 N error.

In our experiments, the frictional force tangent to the phantom surface, i.e., along the *z*-axis, is directed toward the manipulator’s straight incompressible backbone and hence unobservable by the proposed method. In theory, this value can be estimated based on the identified coefficient of friction as *f_σ_z__* = *μf_σ_xy__*. We measured the coefficient of the friction between the manipulator surface and the aortic tissue phantom to be 0.25 ± 0.05.

However, our results suggested that the manipulator’s sliding motion against the tissue consists of stick and sleep instances due to the irregularity of the robot and tissue surfaces. This results in force values larger than the predictions based on the lateral forces and the coefficient of friction. To overcome this, the robot should be operated in a slightly bent configuration. Alternatively, such axial forces in the robot’s straight configuration can be measured directly by mounting a force sensor on the manipulator base.

### D Insertion Performance

Fig. 15 presents the comparison between the experimental and observed values for the distributed forces normal to a phantom surface during the different automated insertion experiments, as explained in Section III-B4.

The results show good agreement between the observed values and the real results in all the cases, with an average 0.09 ± 0.08 N error. However, in cases with activated safety force feedback (i.e., b and d), the maximum real forces measured on the tissues are relatively lower (0.65 w.r.t. 1.20 N). In this experiment, we did not evaluate the positioning accuracy.

### E Visual Center Point Estimation

The navigator software was evaluated by a moving target board at various distances. Circles with a 23 mm diameter and three points instead of commissures were printed on the target board. The calculated circles had a 0.5 mm average distance error between the centers and 22.6±0.2 mm in diameter. The navigator was also examined in 36 points by moving the board 6 mm in both the *X*- and *Y*-directions. It resulted in an average 4.8±0.4% error. Also, this method resulted in a 2.3±0.2% error while the board was moved in the *z*-direction.

### F Motion Tracking Performance

Fig. 16 and Table II present the closed-loop control performance, as discussed in Section III-B6, for tracking three trajectories: a planar circle, a curved square, and a planar square. The controller’s trajectory tracking results were compared with those of an open-loop controller based on the presented IM framework. We observed a 54% decrease in the overall trajectory tracking mean error across the three paths, which highlights the need for a closed-loop control scheme.

### G Automated Positioning in the Workspace

The controller and navigator were evaluated in the manipulator’s workspace through ten trails. The average distance between the graphite point and the circles’ center was equal to 2.6±0.8 mm and 1.7% of the robot length.

### H In Vitro Investigation

**Fig. 17:**
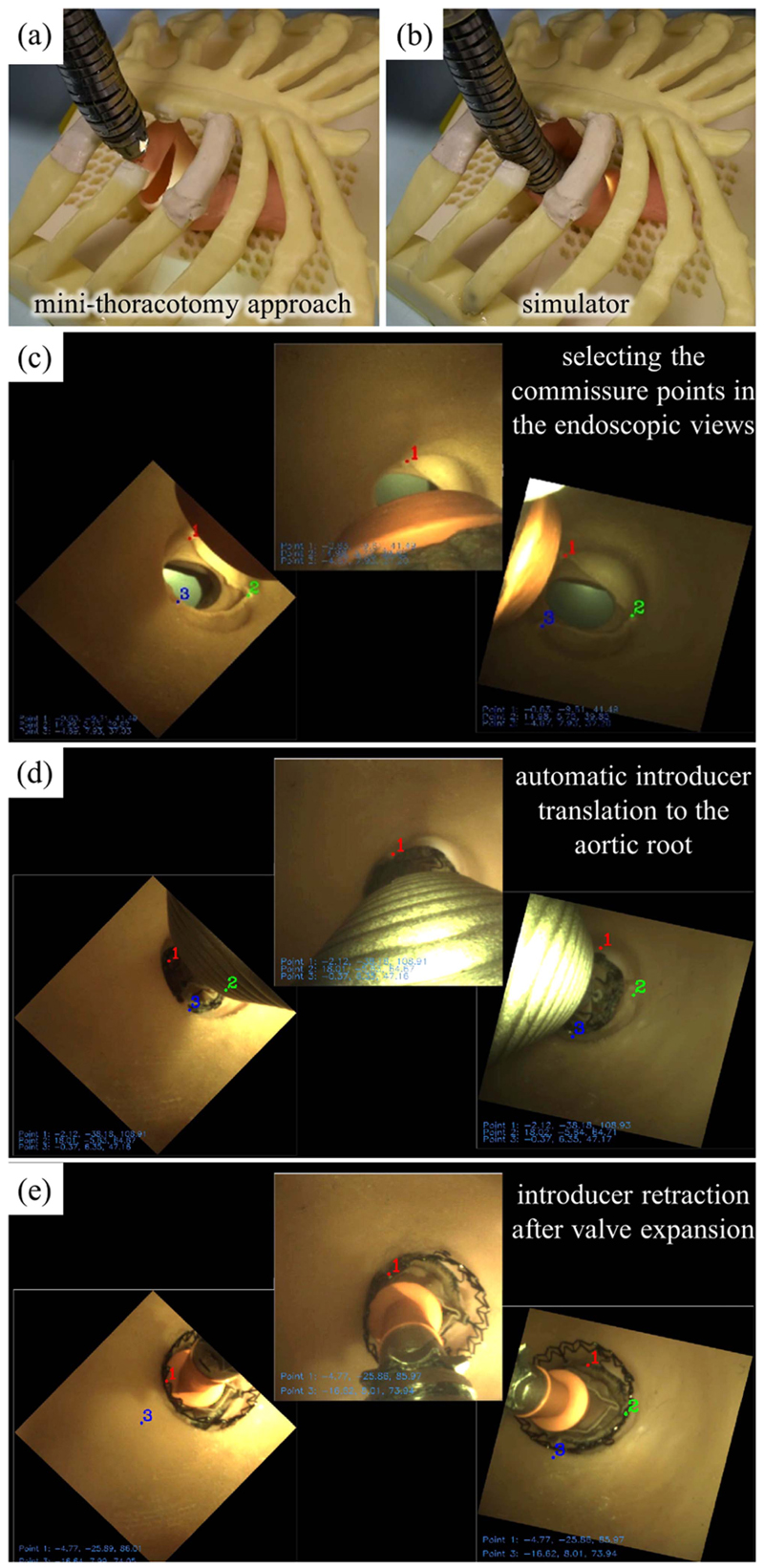
Sorin Perceval S delivery in the simulator following the minithoracotomy approach. (a) and (b) Haptically controlled manipulator while approaching the ascending aorta (Phase I). (c) Camera views and manual anatomical point selection in the surgical navigator (Phase II). (d) Automatic centering of the tip and the introducer translation to the aortic root (Phase III). (e) Introducer retraction from the middle of the expanded valve, ready for the haptically controlled withdrawal.

In simulator valve delivery experiments, the procedure took about 14 min (on average) from valve cartridge preparation to manipulator retraction (see Fig. 17).

Table III presents the misalignment errors in all cases introduced by the three factors described in the last paragraph of Section III-B8.

## V Discussion

The ValveTech robot was designed for Sorin Perceval dimensions in collaboration with two cardiac surgeons, and its functionality was positively validated by surgeons in a previous work in a complete teleoperated framework [28].

The current version of the ValveTech robot underwent major upgrades in its design to facilitate the cameras’ wire movements due to bending. The joysticks were substituted with a haptic interface to guide the manipulator bending more effectively by adding a sense of touch to the system. The controller and the surgical navigator were also linked to autonomously orient the manipulator tip and perform the valve translation. This autonomous control will help surgeons accurately position the valve in a confined space, resulting in less release mismatches.

The system’s FM and IM were developed based on modified CC assumptions and PVW, with the result of predicting the manipulator tip in the presence of body, external, and tendon frictional forces with low computational cost real-time control performance. The model’s accuracy was comparable to that of a more complex dynamic model based on a reduced-order differential dynamics method using the *TMTDyn* package [38]. This is due to the noncollapsible and highly stiff nature of the robot design. Thus, we employed a modified CC model because of the theoretical simplicity, computational performance (2.1 and 3.5 times faster for FM and IM, respectively), and simple formulation of the system IM for hybrid force/position control and observation tasks. Furthermore, a detailed kinematic model was presented to account for the effects of the internal introducer mechanism during the tip-extending motion.

A PID error compensation term was utilized alongside the developed IM model to form a closed-loop control framework for positioning the manipulator based on the surgical navigation data. The controller performance was showcased for tracking trajectories with various planar and 3-D shapes, highlighting the need for the closed-loop control architecture to achieve the desired performance. The presented IM frameworks can be utilized for hybrid force/shape control of the robot tip, as well as safe insertion navigation of the robot regardless of the robot base distance from the surgery site on the patient body.

A novel hybrid FSO algorithm was successfully implemented by utilizing information about the tendon displacement and tension values. The observer algorithm predicted the loads on the manipulator tip with a 3.3% accuracy. This method offers the advantage of estimating the system’s internal and external frictional effects within a short response time, making it suitable for a real-time system. Even though only the force observation accuracy in the global frame *z*-axis direction was evaluated (see Fig. 14), the same principle can be applied for forces in the *x*- and *y*-axis directions.

The distributed force estimation technique successfully estimated the forces between the manipulator body and the phantom during insertion, with an average error of 0.09 N. Moreover, by implementing the safety feature and haptic force feedback, the damages due to insertion were reduced. However, the cases involving automated insertion were similar to manual ones because the phantom curvature was simple to navigate.

The surgical navigator showed very good performance in calculating the landmark coordinates: The annulus center resulted in an average error of 0.5 mm. However, the difficulties involved in manually selecting the commissures in a real operation can increase this error. In fact, the setup for measuring the accuracy of the 3-D center features highly visible markers that uniquely represent the commissures by color difference. In a real surgical scenario, the anatomical landmarks are not significantly different in color from the aortic walls, and only the surgeon’s experience can ensure a reliable selection. Furthermore, the experimental setup consists of a rigid plane, whereas the anatomical commissures reside in a deformable plane, and the aorta motion can contribute to inaccuracies in the estimations. All these additional difficulties are taken into account in the tests for performing artificial valve delivery within the silicon mock-up.

The introducer mechanism can successfully translate the valve by the proposed model. The looseness in the coil attachment and friction in the mechanism imposed different errors in forward–backward motion. The introducer assumes the manipulator bending as an arc, which can be achieved from FK, but sometimes in a surgical scenario, this assumption might not be very accurate, leading to extra error. This error can be compensated by utilizing even a simple PID controller to match the valve and annulus with the help of surgical navigator feedback.

The robotic system was validated in its workspace, incorporating IK and a navigator to touch a center point. The experiment resulted in acceptable errors while all the systems were gathered in a unified controller. The navigator was able to follow the selected points while the target was repositioning slowly, as demonstrated in the experiment. However, the introducer might mislead the selected points during its motion, which need reselection by the surgeon.

Furthermore, the effectiveness of the proposed robot was experimentally confirmed. Positioning accuracy was examined quantitatively in comparison with the ideal valve position. The average distance between centroids was around 2±0.2 mm (first column of Table III), which directly shows our introducer and navigator performance. The average angle between two planes was around 3.4±0.9° (second column of Table III), which can be correlated with the tip angle reaching the surgical site. Moreover, the average rotational error in the aorta normal axis was around 9.8±2.2° (third column of Table III). These errors are mostly related to our ability to precisely recognize the anatomical commissure points from camera images.

The systems reported in the state of the art are not fully characterized to reveal their delivery errors or their validation does not match the real soft aorta anatomy. The straight pneumatic robot presented by Ming et al. showed a 1.14 ± 0.33 mm error in matching centroids based on MRI feedback in a simple tube [16]. robotically-actuated delivery sheath (RADS), presented by Vrooijink et al., showed a mean positioning error of approximately 2 mm along the *x*- and *y*-axes based on ultrasound tracking in a free water container [17].

## VI Conclusion

Robotic-assisted aortic valve replacement has been performed rarely using the da Vinci system [10]. This surgery would be more compatible with da Vinci if dedicated instrumentations (e.g., for holding and releasing the valves) were proposed for the system. Generally, task-specific robotic systems are more cost-efficient and less dependent on learning curves in specialized hospitals. Up to now, only one rigid robot has been designed in previous studies to partially position the heart valve with the help of MRI feedback [16].

In this study, the authors enhanced the ValveTech robot with hybrid FSO, and image-based closed-loop control was introduced to increase the valve positioning accuracy. Its suitability in MIAVR has been proven [28], and new features have been validated here separately in specific experiments. Also, this robot has been validated in terms of positioning accuracy in an artificial chest phantom. In comparison with previous evaluations [28], the new controller resulted in more than a 47% decrease in the average distance between centroids, a 61% decrease in the angle between two planes, and a 39% decrease in rotational error.

The authors utilized surgical navigation based on embedded vision on the tip of manipulator that—in comparison to other visualization means, such as MRI or fluoroscopy—is safer and less bulky. On the other hand, sophisticated flexible endoscopic cameras and image processors might bring higher quality and accuracy to the surgical navigator and valve positioning.

This field of surgery demands very precise alignment of the heart valve with the aortic annulus, as the occurrence of patient prosthesis mismatch results in early and long-term leakages [18]. In addition, a rotational mismatch between the prosthesis and aortic root might result in the blockage of side coronary arteries [39]. Possibly, other equipment or dedicated sensors could be necessary for even more accurate positioning.

In future developments, the manipulator can be covered with a force-sensitive sleeve to achieve more accurate shape observation accuracy and to provide richer haptic feedback. Further validations will include autonomous landmark detection in the surgical navigator, learning curves for valve delivery, and more trials with anatomies closer to humans, such as human cadavers or animals’ aortic root with adequate diameter (e.g., around 23 mm).

## Supplementary Material

supp1-3315966

Appendix

## Figures and Tables

**Table I:** Shape Estimation Error Analysis (in mm) and Calculation Frequency (in Hz) for FM, IM, and FSO Frameworks in Comparison to Experimental Scenario 1–3 (see Figs. 9–13)

	Case 1 (515 points)	Case 2 (408 points)	Case 3 (1500 points)	Average per No. of Points
**FM**	10 mm 6.3 % 71 Hz	16.1 mm 10% 71 Hz	18.9 mm 11.9% 101 Hz	16.5 mm 10.4% 89 Hz
**IM**	Not analyzed	0.3 mm 0.2 % 39 Hz	0.8 mm 0.5 % 54 Hz	0.7 mm 0.4 % 51 Hz
**FSO**	Not analyzed	16.1 mm 10% 22 Hz	18.9 mm 11.9% 30 Hz	18.2 mm 11.5% 28 Hz

Note: Shape % error is w.r.t. the robot length.

**Table II:** Error Analysis (in mm and %) for Trajectory Tracking Based on the Proposed Open-Loop (OL) Controller With the System IM, the Closed-Loop (CL) Controller With a PID Term (see Fig. 15), and the CL Controller With a Load (CL+L)

	Planar Circle	Curved Square	Planar Square
**OL**	3.4 mm 2.1 %	5.3 mm 3.3 %	9.1 mm 5.7 %
**CL**	1.2 mm 0.7 %	2.3 mm 1.4%	5.7 mm 3.5 %
**CL+L**	1.3 mm 0.8 %	2.5 mm 1.5%	6.3 mm 3.9 %

**Table III:** Valve Release Misalignment Results

Trial number	d [mm]	α [degree]	β [degree]
**Trial 1**	1.8	3.5	10.2
**Trial 2**	2.2	5.0	7.6
**Trial 3**	2.4	3.4	6.4
**Trial 4**	2.3	3.8	14.5
**Trial 5**	2.0	2.0	10.2
**Trial 6**	2.1	3.5	11.6
**Trial 7**	2.0	4.2	8.7
**Trial 8**	1.9	3.4	10.2
**Trial 9**	1.8	2.2	8.3
**Trial 10**	2.0	2.8	10.3
**Average**	2.0±0.2	3.4±0.9	9.8±2.2

Note: *d* is the distance between two centroids; *α* is the intersecting angle of two planes; and *β* is the average angle between each pair of three vertices.

## Footnotes

1 Infinite possible *ϕ* values based on the IK formulation when the robot has zero curvature.

## References

[1] Joseph J, Naqvi SY, Giri J, Goldberg S (2017). Aortic stenosis: Pathophysiology, diagnosis, and therapy. Amer J Med.

[2] Czarny MJ, Resar JR (2014). Diagnosis and management of valvular aortic stenosis. Clin Med Insights Cardiol.

[3] Paparella D (2017). Minimally invasive heart valve surgery: Influence on coagulation and inflammatory response. Interactive Cardiovasc Thoracic Surg.

[4] Shehada SE (2017). Minimal access versus conventional aortic valve replacement: A meta-analysis of propensity-matched studies. Interactive Cardiovasc Thoracic Surg.

[5] Glauber M, Ferrarini M, Miceli A (2015). Minimally invasive aortic valve surgery: State of the art and future directions. Ann Cardiothoracic Surg.

[6] Bonow RO, Leon MB, Doshi D, Moat N (2016). Management strategies and future challenges for aortic valve disease. Lancet.

[7] Grigorios T (2018). Transcatheter versus surgical aortic valve replacement in severe, symptomatic aortic stenosis. J Geriatr Cardiol.

[8] Folliguet TA, Vanhuyse F, Konstantinos Z, Laborde F (2005). Early experience with robotic aortic valve replacement. Eur J Cardio-Thoracic Surg.

[9] Suri RM, Burkhart HM, Schaff HV (2010). Robot-assisted aortic valve replacement using a novel sutureless bovine pericardial prosthesis: Proof of concept as an alternative to percutaneous implantation. Innovations, Technol Techn Cardiothoracic Vasc Surg.

[10] Balkhy HH, Lewis CTP, Kitahara H (2018). Robot-assisted aortic valve surgery: State of the art and challenges for the future. Int J Med Robot Comput Assist Surg.

[11] Vola M (2016). Robotic total endoscopic sutureless aortic valve replacement: Proof of concept for a future surgical setting. Int J Med Robot Comput Assist Surg.

[12] Sun J (2022). Early results of totally endoscopic robotic aortic valve replacement: Analysis of 4 cases. J Cardiothoracic Surg.

[13] Wei LM, Cook CC, Hayanga JWA, Rankin JS, Mascio CE, Badhwar V (2022). Robotic aortic valve replacement: First 50 cases. Ann Thoracic Surg.

[14] Rodriguez E (2016). Pathway for surgeons and programs to establish and maintain a successful robot-assisted adult cardiac surgery program. J Thoracic Cardiovasc Surg.

[15] Freschi C, Ferrari V, Melfi F, Ferrari M, Mosca F, Cuschieri A (2013). Technical review of the da Vinci surgical telemanipulator. Int J Med Robot Comput Assist Surg.

[16] Li M, Kapoor A, Mazilu D, Horvath KA (2011). Pneumatic actuated robotic assistant system for aortic valve replacement under MRI guidance. IEEE Trans Biomed Eng.

[17] Vrooijink GJ, Ellenbroek TTM, Breedveld P, Grandjean JG, Misra S (2014). A preliminary study on using a robotically-actuated delivery sheath (RADS) for transapical aortic valve implantation.

[18] Gilmanov D (2013). Sutureless implantation of the perceval s aortic valve prosthesis through right anterior minithoracotomy. Ann Thoracic Surg.

[19] Merk DR (2011). Image-guided transapical aortic valve implantation sensorless tracking of stenotic valve landmarks in live fluoroscopic images. Innovations, Technol Techn Cardiothoracic Vasc Surg.

[20] Karar ME, Merk DR, Falk V, Burgert O (2016). A simple and accurate method for computer-aided transapical aortic valve replacement. Computerized Med Imag Graph.

[21] Wu K (2019). Safety-enhanced model-free visual servoing for continuum tubular robots through singularity avoidance in confined environments. IEEE Access.

[22] Wang X (2020). Eye-in-hand visual servoing enhanced with sparse strain measurement for soft continuum robots. IEEE Robot Automat Lett.

[23] Russoetal M (2023). Continuum robots: An overview. Adv Intell Syst.

[24] Concistrè G (2018). Aortic valve replacement with perceval bioprosthesis: Single-center experience with 617 implants. Ann Thoracic Surg.

[25] Lorusso R, Gelsomino S, Renzulli A (2013). Sutureless aortic valve replacement: An alternative to transcatheter aortic valve implantation?. Curr Opin Cardiol.

[26] Tamadon I, Soldani G, Dario P, Menciassi A (2018). Novel robotic approach for minimally invasive aortic heart valve surgery.

[27] Tamadon I, Huan Y, Groot AG, Menciassi A, Sinibaldi E (2020). Positioning and stiffening of an articulated/continuum manipulator for implant delivery in minimally invasive surgery. Int J Med Robot Comput Assist Surg.

[28] Tamadon I (2021). ValveTech: A novel robotic approach for minimally invasive aortic valve replacement. IEEE Trans Biomed Eng.

[29] Klein P, Klop IDG, Kloppenburg GLT, van Putte BP (2018). Planning for minimally invasive aortic valve replacement: Key steps for patient assessment. Eur J Cardio-Thoracic Surg.

[30] Erbel R, Eggebrecht H (2006). Aortic dimensions and the risk of dissection. Heart.

[31] Sadati SMH, Naghibi SE, Shiva A, Walker ID, Althoefer K, Nanayakkara T (2017). Mechanics of continuum manipulators, a comparative study of five methods with experiments.

[32] Webster RJ, Jones BA (2010). Design and kinematic modeling of constant curvature continuum robots: Areview. Int J Robot Res.

[33] Mamone V (2019). Low-computational cost stitching method in a three-eyed endoscope. J Healthcare Eng.

[34] Gallup D, Frahm J-M, Mordohai P, Pollefeys M (2008). Variable baseline/resolution stereo.

[35] Mamone V, Di Fonzo M, Esposito N, Ferrari M, Ferrari V (2020). Monitoring wound healing with contactless measurements and augmented reality. IEEE J Transl Eng HealthMed.

[36] Karbasizadeh N, Zarei M, Aflakian A, Masouleh MT, Kalhor A (2018). Experimental dynamic identification and model feed-forward control of Novint Falcon haptic device. Mechatronics.

[37] Turini G (2017). Augmented Reality, Virtual Reality, and Computer Graphics.

[38] Sadati SMH (2021). TMTDyn: A Matlab package for modeling and control of hybrid rigid–continuum robots based on discretized lumped systems and reduced-order models. Int J Robot Res.

[39] Wollersheim LW, Li WW, De Mol BA (2014). Current status of surgical treatment for aortic valve stenosis. J Cardiac Surg.

