# Supplementary material for: Semiautonomous Robotic Manipulator for Minimally Invasive Aortic Valve Replacement
Source: IEEE Trans Robot. Author manuscript; Available in PMC 2024 Jan 20. (PMC7615540; doi:10.1109/TRO.2023.3315966)
Supplement: Appendix [file EMS192638-supplement-Appendix.pdf]

APPENDIX A
CC FORWARD AND INVERSE MECHANICS

A. FK Based on CC Assumptions

Assuming the bending axis is the y-axis in the bending plane, the transformation for the manipulator tip T_M fixed frame can be found based on a series of four consecutive transformation for

1) a rotation of ϕ around the reference frame z-axis to align the local frame y-axis with the bending axis, T_ϕ,
2) a translation from the bending plane origin to the manipulator tip location, T_ρ,
3) a rotation of $\theta = \kappa l_M$ around the bending axis (i.e., local frame y-axis), T_θ,
4) a correction rotation around the local z-axis of $-\phi$ to align the local frame with the manipulator tip material frame, $T_{-\phi} = T_\phi^T$.

The introducer tip frame is derived by considering a transformation of l_I along the manipulator tip local frame z-axis, T_I. The overall transformation to points on the robot manipulator T_M and introducer T_T is derived based on the postmultiplication rule as follows:

$$\begin{aligned} T_M &= T_\phi \cdot T_\rho \cdot T_\theta \cdot T_{-\phi}, \\ T_T &= T_\phi \cdot T_\rho \cdot T_I \cdot T_\theta \cdot T_{-\phi}, \end{aligned} \tag{25}$$

where

$$\begin{aligned} T_\phi &= \begin{bmatrix} \cos(\phi) & -\sin(\phi) & 0 & 0 \\ \sin(\phi) & \cos(\phi) & 0 & 0 \\ 0 & 0 & 1 & 0 \\ 0 & 0 & 0 & 1 \end{bmatrix}, \\ T_\rho &= \begin{bmatrix} 1 & 0 & 0 & (1-\cos(\theta))/\kappa \\ 0 & 1 & 0 & 0 \\ 0 & 0 & 1 & \sin(\theta)/\kappa \\ 0 & 0 & 0 & 1 \end{bmatrix}, \\ T_\theta &= \begin{bmatrix} \cos(\theta) & 0 & \sin(\theta) & 0 \\ 0 & 1 & 0 & 0 \\ -\sin(\theta) & 0 & \cos(\theta) & 0 \\ 0 & 0 & 0 & 1 \end{bmatrix}, \\ T_I &= \begin{bmatrix} 1 & 0 & 0 & l_I \cos(\pi/2-\theta) \\ 0 & 1 & 0 & 0 \\ 0 & 0 & 1 & l_I \sin(\pi/2-\theta) \\ 0 & 0 & 0 & 1 \end{bmatrix}. \end{aligned} \tag{26}$$

APPENDIX B

The FM for shape observation and a hypothetical hybrid shape control and force observer framework are discussed in this Appendix.

A. Forward Mechanics for Shape Observation

In an FM framework, $[\kappa_x, \kappa_y]$ are unknown in the $\bar{w}_{,\kappa_{xy}}$ relation. A numerical solution can be sought for the system states given the tendon lengths l_{P_i} [as in (12)] and based on the CC IK solution $[\kappa_{x_{\mathrm{CC}}}, \kappa_{y_{\mathrm{CC}}}]$ as a close enough initial guess for faster convergence of the numerical solver.

B. Hybrid Shape Control and Force Observer

Similar to a hybrid FSO, we can formulate a hybrid shape control and force observer to control the robot tip position while estimating the external forces at the introducer or manipulator tip. To this end, we used a combination of the relations for $w_{KP,q} = w_{K_{,q}} - w_{P_{,q}}$ and $w_{,q}$ while considering $[\kappa_x, \kappa_y]$ as known variables [based on the desired robot curvature and bending direction $[\kappa^*, \phi^*]$ or tip trajectory ρ_T^* and the IK map in (2) and (3)] and $[\kappa_{x0}, \kappa_{y0}, l_I]$, as well as one of the following sets $[f_{F_x}, f_{F_y}, f_{F_z}]$, $[f_{\sigma_x}, f_{\sigma_y}, f_{\sigma_z}]$, or $[p_{\mu_x}, p_{\mu_y}]$, as the unknowns. The results for the different discussed mechanics maps are presented in Section IV.
